# On springtails (Hexapoda: Collembola): a morphofunctional study of the jumping apparatus

**DOI:** 10.1186/s12983-022-00463-y

**Published:** 2022-07-29

**Authors:** Fábio Gonçalves de Lima Oliveira

**Affiliations:** Institut Für Biowissenschaften, Universität Rostock, Allgemeine und Spezielle Zoologie, Universitätsplatz 2, 18055 Rostock, Germany

**Keywords:** Basal plates, Basal sclerites, Furca, Hemolymph pressure, Resilin, Retinaculum, Spring mechanism

## Abstract

**Background:**

Springtails (Hexapoda: Collembola) are tiny organisms that lead a hidden life, mostly occuring deep in the soil and on leaf litter. They have a variety of interesting body morphology patterns, the most famous of which is the catapult-like structure that enables them to jump and flee from predators. This highly specialized jumping apparatus consists of a mobile furca, which when at rest fits into a trigger, "the retinaculum" on the ventral side of the abdomen. Despite the many studies that have attempted to investigate the jumping apparatus, the actual mechanisms involved in the jump, for example the way in which the furca is released by the retinaculum, how and where the mechanisms of spring and hydrostatic pressure originate, are still not properly understood. The morphology of the jumping apparatus of *Orchesella cincta* was investigated in detail using confocal laser scanning microscopy and MicroCT techniques for 3D reconstruction.

**Results:**

The morphology of *O. cincta* with both flexed and extended furca is analysed and described. The abdominal musculature involved in the jumping mechanism and relevant structures of the exoskeleton of retinaculum and furca are described in detail. With the data obtained in this study, hypotheses can be made about (1) where and how the spring and hydrostatic pressure mechanisms originate; (2) which muscles act on the extension and flexion of the furca; (3) which muscles act on the retinaculum and (4) how the retinaculum is released from the furca.

**Conclusions:**

The comparative morphological study proved informative, and shows how springtail jumping involves mechanisms unique to this taxon. Hydrostatic pressure regulation possibly varies between animals with distinct segmentation, and those with fused segmentation. Interesting cuticular characters were revealed, such as basal plates and sclerites related to the construction of the spring mechanism. The present study establishes itself as a model option for future morphofunctional studies on springtail’s jumping. Analysis of videos and images using a high speed camera will be useful for understanding how the jump develops through take-off, aerial and landing phases.

**Supplementary Information:**

The online version contains supplementary material available at 10.1186/s12983-022-00463-y.

## Background

Small animals like arthropods that perform fast movements such as predation and jumping depend on much greater limb accelerations than larger animals. To overcome the temporal limitations of muscle contraction, some arthropods developed, independently, a strategy for power amplification, the spring mechanism involving motors and latches. The spring mechanism enables the animal to store energy for the desired movement and release it instantly when needed [1–3].

**Fig. 1:**
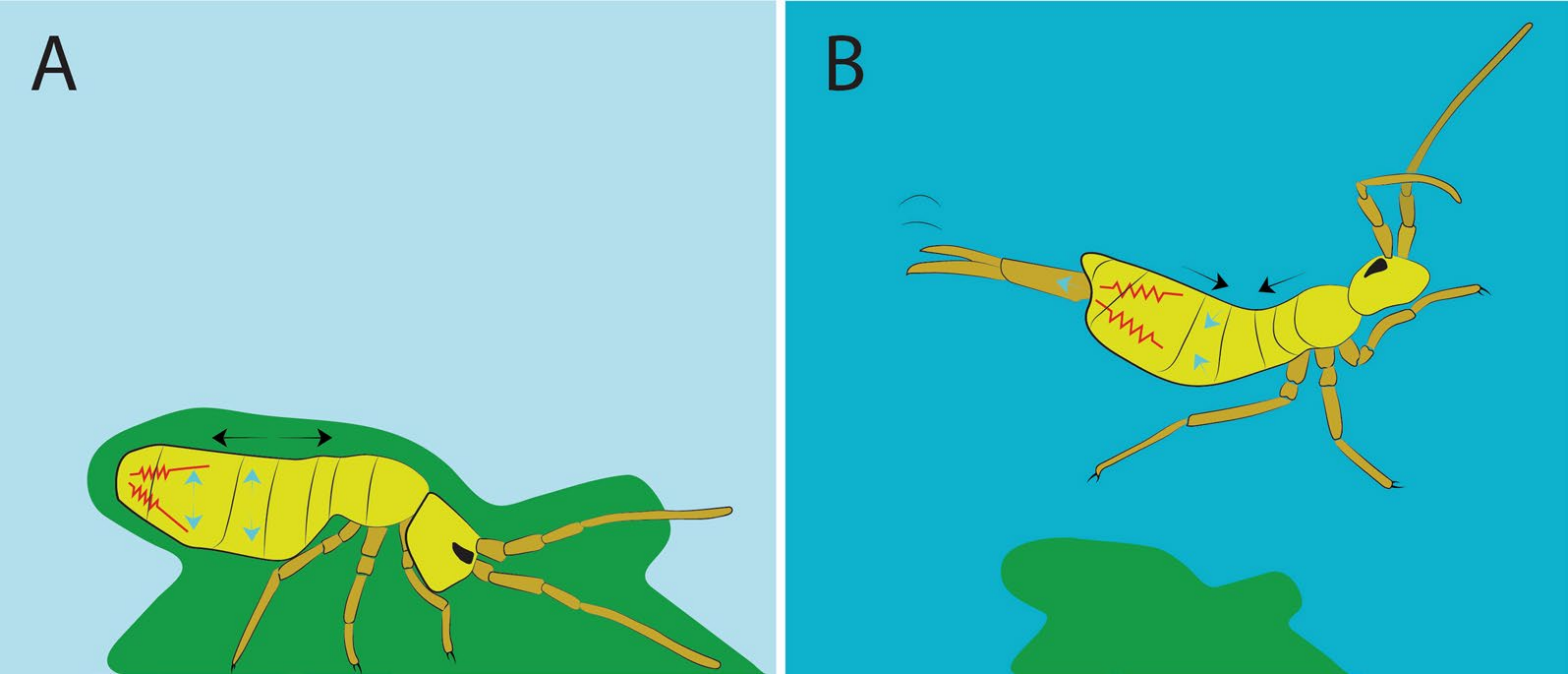
Schematic image of springtails jumping. **A** Furca flexed at rest, spring contracted; **B** Furca extended during jump, spring relaxed. Black arrows show the contour of the segments. Blue arrows show hydrostatic flow. Red springs illustrate the spring mechanism concept

Springtails are tiny hexapods (Collembola) (0.1–5.0 mm) that predominantly inhabit the surface layer of the ground and are commonly found on/among litter fragments [4–7]. One of the most remarkable features of these animals is their ability to jump in order to escape from predators, a feat only made possible by a highly sophisticated device (Fig. 1) [8, 9]. Describing it superficially, this apparatus is essentially composed of a propulsion organ, the furca, and a retinaculum where the furca is held until the moment of the jump.

Although the various mechanisms involved in the springtail jump have been addressed in previous studies, due to the scarcity of morphological evidence there are still questions which remain to be clarified. The muscular system in segmented springtails was described by Manton [10] and Eisenbeis [11], both studies using *Tomocerus longicornis* (Müller, 1776). Manton [10] offered a morphofunctional interpretation of the jumping, while Eisenbeis [11] took a more descriptive approach. Manton [10], Eisenbeis and Ulmer [12] and Christian [13] all mentioned the basal sternites (abdominal segments 4-6th) as important parts of the jumping apparatus, due to their mobility and elasticity. Manton [10] and Christian [13] described the basal sclerite of the 4th abdominal segment, as the “basal rods”, hypothesizing that the elastic energy required for the spring mechanism could be stored there. The mechanism that triggers the jump (releasing the spring) is the subject of a discussion that has not yet been resolved. Manton [10] proposed that hydraulic pressure alone triggers the jump, but this was later refuted by Christian [13], who believed the release of the spring was mainly the result of muscular action. Eisenbeis and Ulmer [12] and Brackenbury and Hunt [14] agreed that the two mechanisms (muscle system and hydraulic pressure) could act together or independently as a motive force to release the spring. However, our understanding of how and where these two different mechanisms (spring and hydraulic pressure) originate on a body systematic level is still preliminary.

The retinaculum and furca are, respectively, modified states of the ambulatory legs of the 3rd and 4th abdominal segments [15] which diversified more than 400 MA [16] into these two very unique structures that work together in an intersegmental relationship to facilitate the famous springtail jump. In addition to being the oldest, springtails are also the most abundant and widely distributed hexapods on the planet, occurring in most strata (horizontal and vertical distribution in the landscape). Their habitats include the forest canopy and soil surface, deeper layers of the soil, the surface of lakes, and even your home. As the soil depth at which they are found increases, a reduction in body size and a shortening of legs, antennae and furca can be observed (these can be also absent due to secondary loss) [17]. A wide range of body shapes are noted among springtails, according to the environment in which they live. At the same time, a high degree of convergence means that similarly shaped structures are found in taxa that do not have a close phylogenetic connection [18]. This diversity of morphological shapes and habitat use, the miniaturized body architecture [19] and the presence of appendages exclusively used in jumping make springtails one of the most interesting model organisms for the study of spring mechanisms and jumping behavior in Arthropoda.

In this study, which uses confocal laser scanning microscopy (cLSM), MicroCT and 3D models, I describe the jumping apparatus of *Orchesella cincta* (Linnaeus, 1758), including the muscular system of the abdomen, retinaculum and furca, and cuticular structures such as the tergites, basal sternites and elastic endosclerites of the abdomen, retinaculum and furca. This is the first comparative investigation using 3D reconstructions into morphological shape in the "flexed furca" and "extended furca" phases, and it culminates in an interpretation and discussion of the morphofunctional mechanisms of the jump showing the main elements behind this phenomenon and explaining how and where the hydraulic pressure and spring mechanism potentially originate.

## Results

**Fig. 2:**
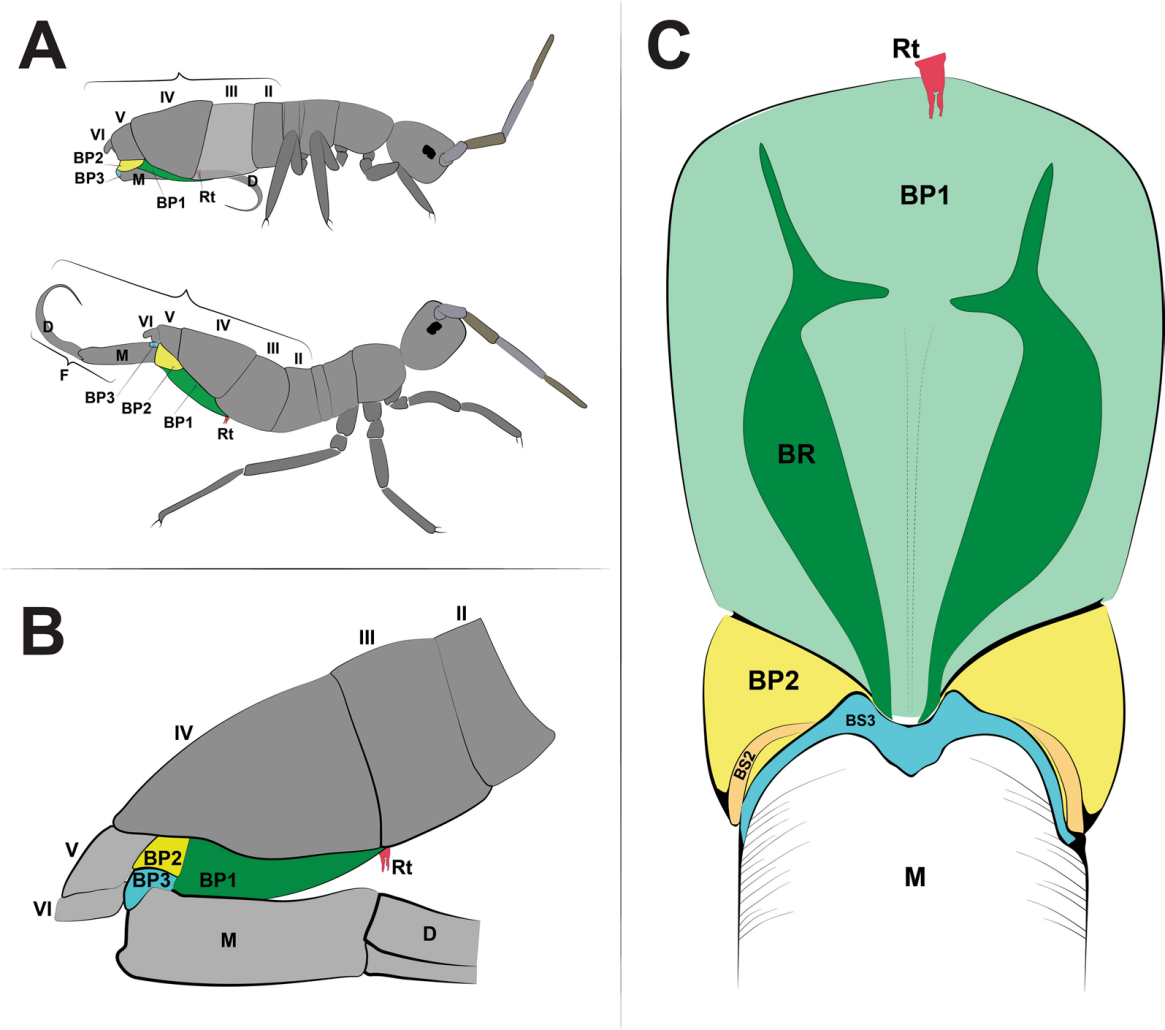
Morphofunctional study of the jumping apparatus in *Orchesella cincta*. **A** The reconstructed abdominal segments and a comparison between flexed and extended furca phases. **B** Lateral view of the jumping apparatus, comprised of abdominal segments 2nd–6th. **C** Ventral view of the jumping apparatus— adapted from Manton [10] on *Tomocerus longicornis* (Müller, 1776). BP1: Basal plate 1, BP2: Basal plate 2; BP3: Basal plate 3; M: Manubrium; F: Furca; D: Dens; Rt: Retinaculum; BR: Basal rods; BS2: Basal sclerite 2; BS3: Basal sclerite 3

**Fig. 3:**
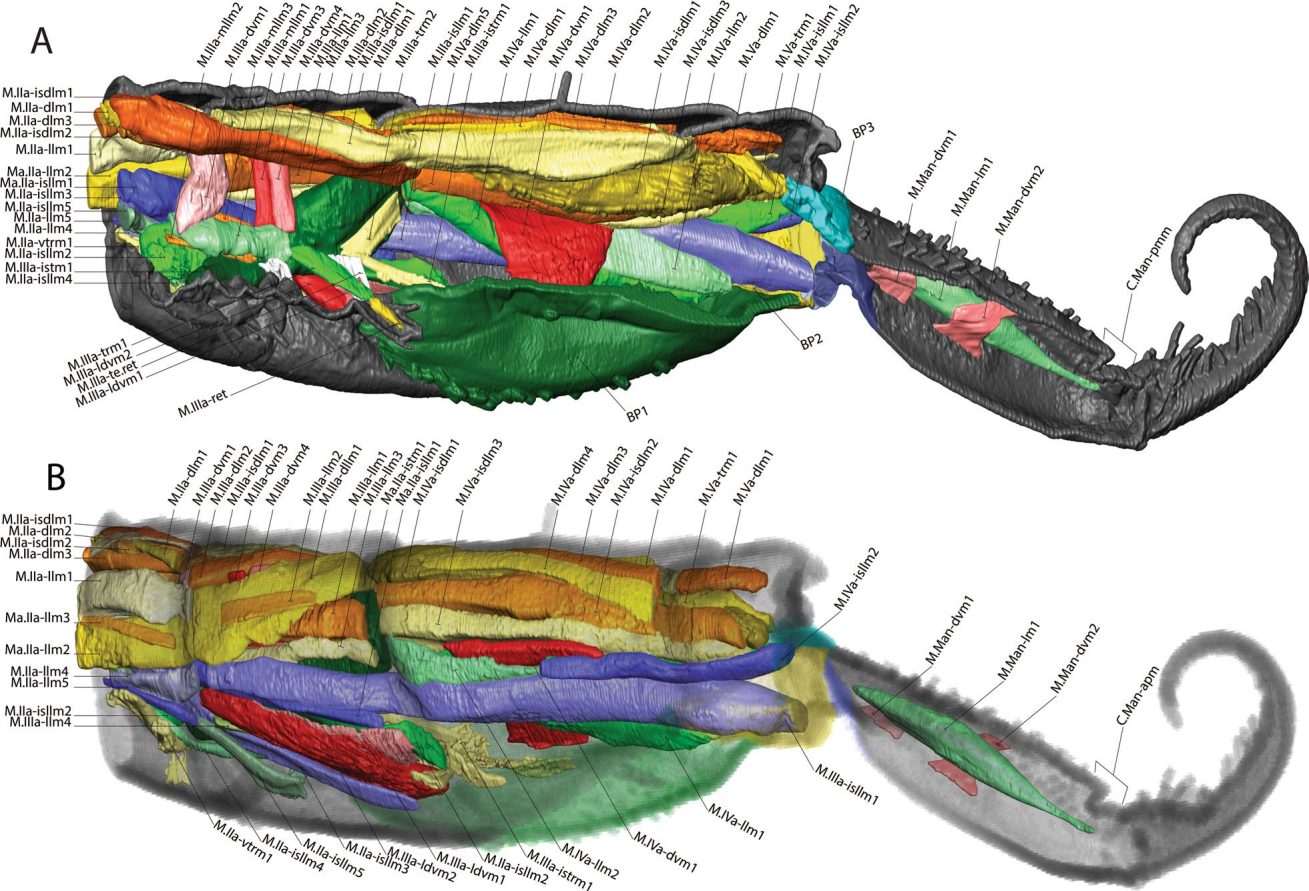
MicroCT morphological reconstruction of the 2nd–6th abdominal segments, the jumping apparatus, cuticle and musculature in *Orchesella cincta* with the furca extended. **A** Internal lateral view of the jumping apparatus (furca extended). **B** External lateral view of the jumping apparatus (furca extended)

**Fig. 4:**
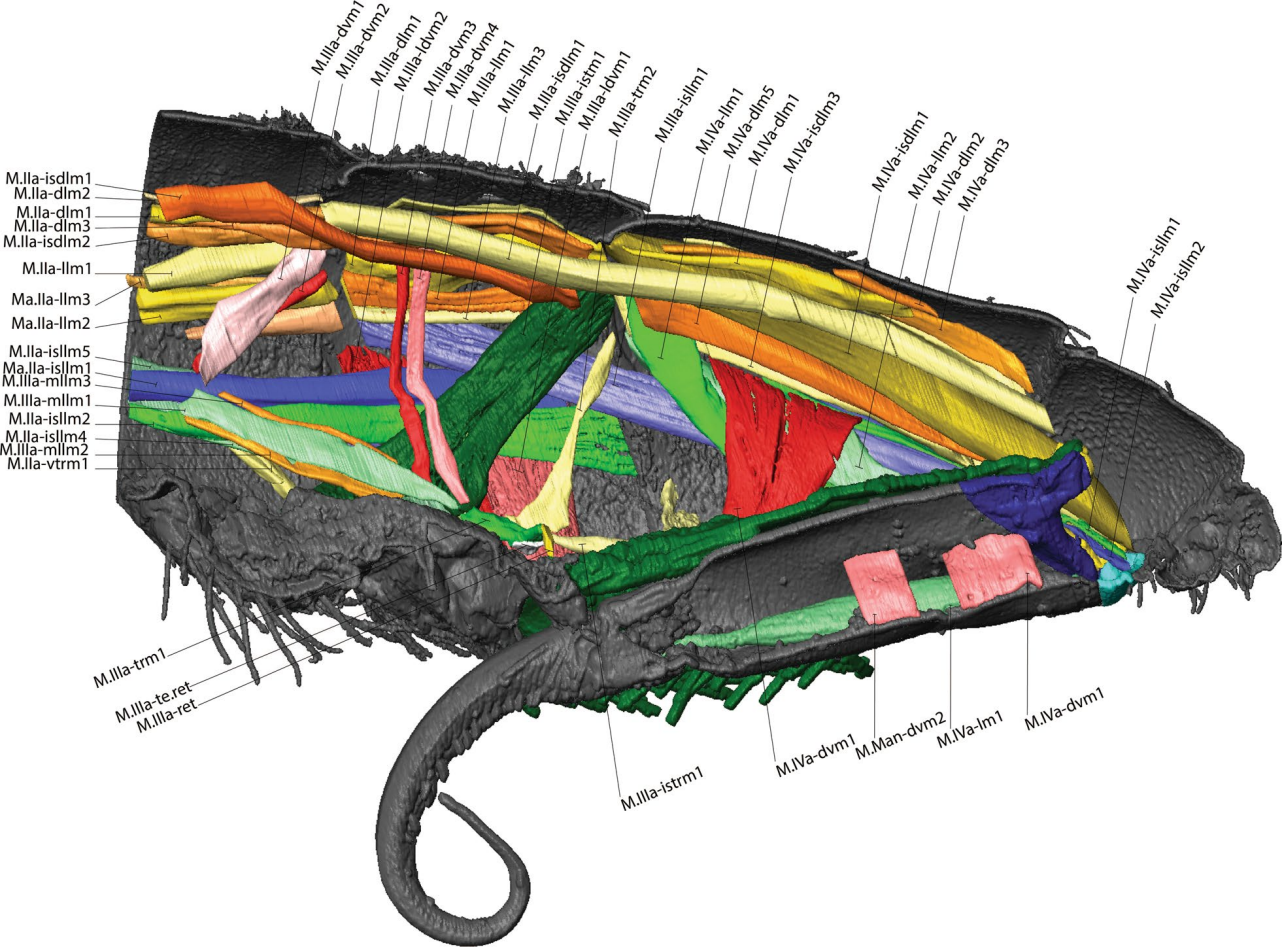
MicroCT morphological reconstruction of the 2nd–6th abdominal segments, the jumping apparatus, cuticle and musculature in *Orchesella cincta* with the furca flexed. Internal lateral view of the jumping apparatus (furca flexed)

**Fig. 5:**
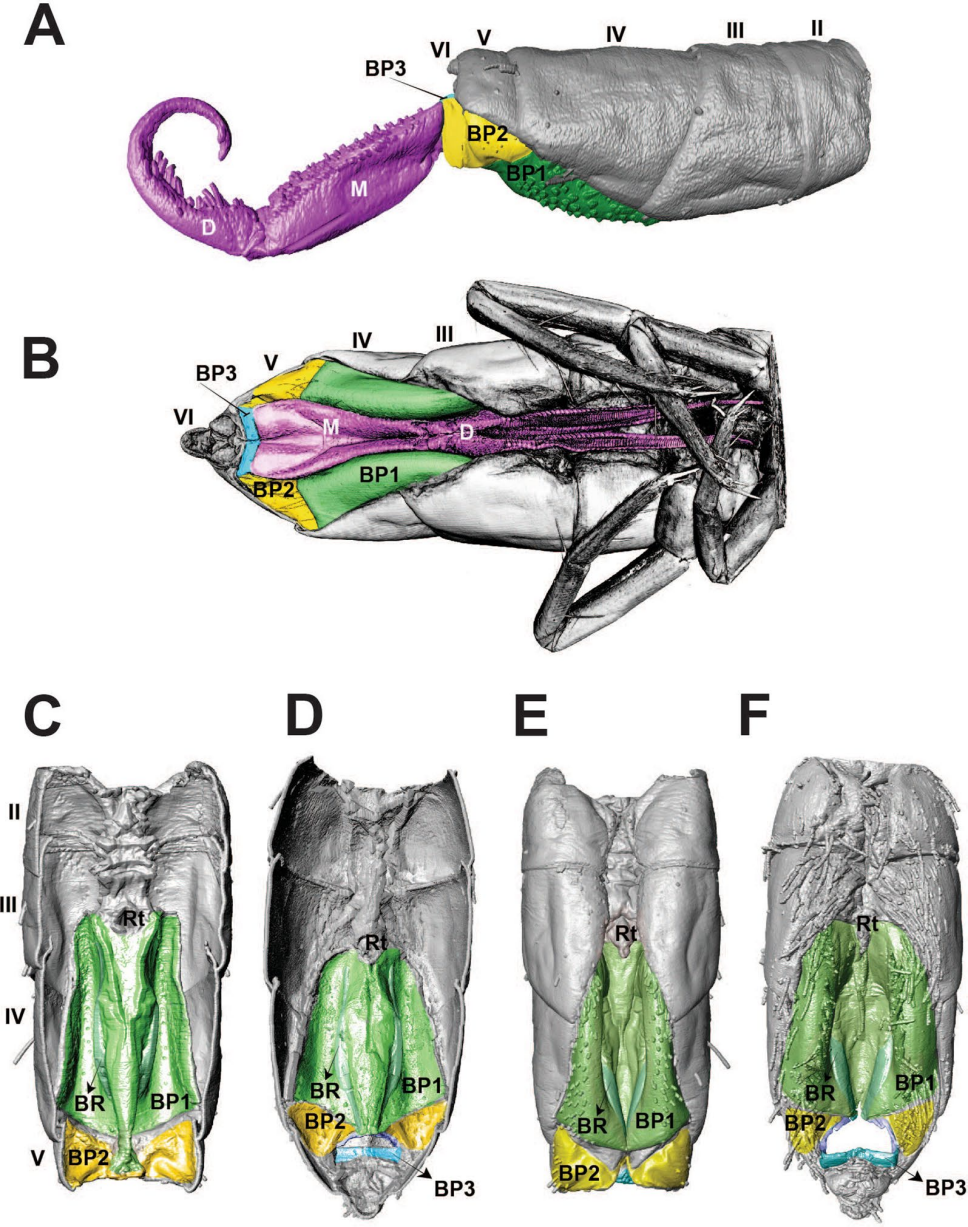
MicroCT morphological reconstruction of the Basal plates of *Orchesella cincta*. **A** Lateral view of the jumping apparatus (furca extended). **B** Ventral view of the jumping apparatus (furca flexed). Sternites and basal plates. **C**, **E** Furca extended. **D** Furca flexed in dorsal view. **F** Furca flexed in ventral view (furca removed). F) BP1: basal plate 1, BP2: basal plate 2; BP3: basal plate 3; M: manubrium; D: dens; Rt: retinaculum; BR: basal rods

The morphology of the jumping apparatus was studied comparatively between specimens of *Orchesella cincta* with extended and flexed furca (Fig. 2). Abdominal segments 2nd–6th were reconstructed, including cuticular structures such as basal plates and sclerites, as well as internal musculature. The architecture of the abdomen in *O. cincta* is defined by visible and clear segmentation. The 4th abdominal segment is longer than the others, with a well-developed furca which inserts ventrally. A complex muscular system with muscles oriented predominantly parallel to the longitudinal axis is characteristic in *O. cincta*. Recognizable at the base of the furca are the basal plates—mobile and elastic cuticular structures via which the furca articulates with the abdomen on a longitudinal axis. Three basal plates BP1, BP2 and BP3 are found ventrolaterally in the abdomen, connecting to each other at the edges. Their architecture, mobility and elasticity are intrinsically related to the jumping behavior. The movement of the basal plates and the abdominal segments occurs mainly by muscular action. The furca is extended via an anteroposteriorly oriented rotary movement capable of a 180° execution angle. When extended, the furca protrudes posteriorly and is highly exposed. When flexed, the furca folds ventrally together with the basal plates, hiding in the inner part of the abdomen while attached to the retinaculum (Figs. 3A, B, 4, 5A, B).

In the following, the form and functioning of the main parts of the jumping apparatus are described. An interpretation of the morphofunctional mechanisms is provided on the basis of a comparison between individuals with flexed and extended furca. MicroCT reconstructions of all muscles and the cuticular walls of the 2nd–6th abdominal segments can be found in Figs. 3A, B and 4. A descriptive list of the reconstructed muscles and information about attachment points is found in Table 1.

**Table 1:** The muscles of 2nd–6th abdominal segments and their respective attachment points in *Orchesella cincta*

Name of the muscle	Origin	Insertion point
M.IIa-dlm1	Tergite of IIa anterior dorsal medial	Tergite of IIIa anterior dorsal medial
M.IIa-dlm2	Tergite of IIa anterior dorsal medial	Tergite of IIIa anterior dorsal medial
M.IIa-dlm3	Tergite of IIa anterior dorsal medial	Tergite of IIIa anterior dorsal medial
M.IIa-llm1	Tergite of IIa anterior lateral medial	Tergite of IIIa anterior lateral medial
M.IIa-llm2	Tergite of IIa anterior lateral medial	Tergite of IIIa anterior lateral medial
M.IIa-llm3	Tergite of IIa anterior lateral medial	Tergite of IIIa anterior lateral medial
M.IIa-llm4	Tergite of IIa anterior lateral medial	Tergite of IIIa anterior lateral medial
M.IIa-llm5	Tergite of IIa anterior lateral medial	Tergite of IIIa anterior lateral medial
M.IIa-isdlm1	Tergite of IIa anterior dorsomedial	Dorsolateral in transition area between IIIa and IVa
M.IIa-isdlm2	Tergite of IIa anterior dorsomedial	Dorsolateral in transition area between IIIa and IVa
M.IIa-isllm1	Tergite of IIa anterior ventral lateral	Tergite of IIIa ventral lateral in transition area between IIIa and IVa
M.IIa-isllm2	Tergite of IIa anterior ventral lateral	Tergite of IIIa ventral lateral in transition area between IIIa and IVa
M.IIa-isllm3	Tergite of IIa anterior ventral lateral	Tergite of IIIa ventral lateral in transition area between IIIa and IVa
M.IIa-isllm4	Tergite of IIa anterior ventral lateral	Tergite of IIIa ventral lateral in transition area between IIIa and IVa
M.IIa-isllm5	Tergite of IIa anterior ventral lateral	Tergite of IIIa ventro medial in transition area between basal plate 1 and tergite IIIa
M.IIa-vtrm1	Tergite of IIa anterior ventral lateral	Sternite of IIa medial
M.IIIa-mllm1	Muscle center central lateral in transition area of IIa and IIIa	Muscle center central lateral IIIa
M.IIIa-mllm2	Muscle center central lateral in transition area of IIa and IIIa	Muscle center central lateral IIIa
M.IIIa-mllm3	Muscle center central lateral in transition area of IIa and IIIa	Muscle center central lateral IIIa
M.IIIa-istm1	Inner side of the sternite III	Muscle center dorsolateral in transition area between IIIa and IVa
M.IIIa-dvm1	Muscle center central lateral IIIa	Tergite of IIIa mediolateral
M.IIIa-dvm2	Muscle center central lateral IIIa	Tergite of IIIa mediolateral
M.IIIa-dvm3	Muscle center central lateral IIIa	Tergite of IIIa mediolateral
M.IIIa-dvm4	Muscle center central lateral IIIa	Tergite of IIIa mediolateral
M.IIIa-trm1	Muscle center central lateral IIIa	Linked to M.IIIa-ret
M.IIIa-trm2	Muscle center central lateral IIIa posterior	Dorsal lateral in transition area between IIIa and IVa
M.IIIa-istrm1	Muscle center central lateral IIIa posterior	Basal plate 1 mediolateral anterior
M.IIIa-isdlm1	Tergite of IIIa, anterior dorsal medial	Tergite of Va, anterior dorsal medial in transition area between IVa und Va
M.IIIa-isllm1	Laterally in transition area between IIa and IIIa	Sternite of IVa posterolateral in BS3
M.IIIa-ret	Retinaculum internally at the baseof ramus	Linked to M.IIIa-trm1
M.IIIa-llm1	Tergite lateral in transition area between IIa und IIIa	Tergite lateral in transition area between IIIa und IVa
M.IIIa-llm2	Tergite lateral in transition area between IIa und IIIa	Tergite lateral in transition area between IIIa und IVa
M.IIIa-llm3	Tergite lateral in transition area between IIa und IIIa	Tergite lateral in transition area between IIIa und IVa
M.IIIa-llm4	Tergite lateral in transition area between IIa und IIIa	Tergite lateral in transition area between IIIa und IVa
M.IIIa-dlm1	Dorsomedial in transition area between IIa and IIIa	Muscle center dorsomedial in transition area between IIIa and IVa
M.IIIa-dlm2	Dorsomedial in transition area between IIa and IIIa	Muscle center dorsomedial in transition area between IIIa and IVa
M.IIIa-ldvm1	BP1 median lateral point	Tergite IIIa lateral (very long longitudinal point)
M.IIIa-ldvm2	BP1 lateral transition area between BP1 and tergite IIIa	Tergite IIIa lateral (very long longitudinal point)
M.IIIa-te.ret	Muscle center central lateral IIIa	Retinaculum at lateral side of ramus
M.IVa-dlm1	Tergite of IVa anterior dorsal medially	Tergite of Va anterior dorsal medial
M.IVa-dlm2	Tergite of IVa anterior dorsal medially	Tergite of Va anterior dorsal medial
M.IVa-dlm3	Tergite of IVa anterior dorsal medially	Tergite of Va anterior dorsal medial
M.IVa-dlm4	Tergite of IVa anterior dorsal medially	Tergite of Va anterior dorsal medial
M.IVa-dlm5	Tergite of IVa anterior dorsal medially	Tergite of Va anterior dorsal medial
M.IVa-llm1	Tergite of IVa anterior dorsal medially	Ventrally in between the sternites BP1 and BP2
M.IVa-llm2	Tergite of IVa anterior dorsal laterally	Ventrally in between the sternites BP1 and BP2
M.IVa-dvm1	In the BP1, laterally in the basal rod	In the middle of IVa, laterally
M.IVa-isdlm1	Tergite of IVa anterior dorsal medially	Tergite of Va posteriorly in the BP2
M.IVa-isdlm2	Tergite of IVa anterior dorsal medially	Tergite of Va posteriorly in the BP2
M.IVa-isdlm3	Tergite of IVa anterior dorsal medially	Tergite of Va posteriorly in the BP2
M.IVa-isllm1	In the middle of IVa, lateral medially	Tergite of Va posteriorly in the BP3
M.IVa-isllm2	In the middle of IVa, lateral medially	Tergite of Va posteriorly in the BP3
M.Va-trm1	Tergite of Va anterior dorsal medially	Dorsal anteriorly at the BP3
M.Va-dlm1	Tergite of Va anterior dorsal medially	Tergite of VIa anterior dorsal medial
M.Man-dvm1	Ventral medially at the anterior portion of the manubrium	Dorsal medially at the anterior portion of the manubrium
M.Man-dvm2	Ventral medially at the middle portion of the manubrium	Dorsal medially at the middle portion of the manubrium
M.Man-lm1	Basal plate 3 dorsal medially	Laterally at the posterior membrane of manubrium

### Basal plate 1 (BP1) and the basal rod (BR)

**Fig. 6:**
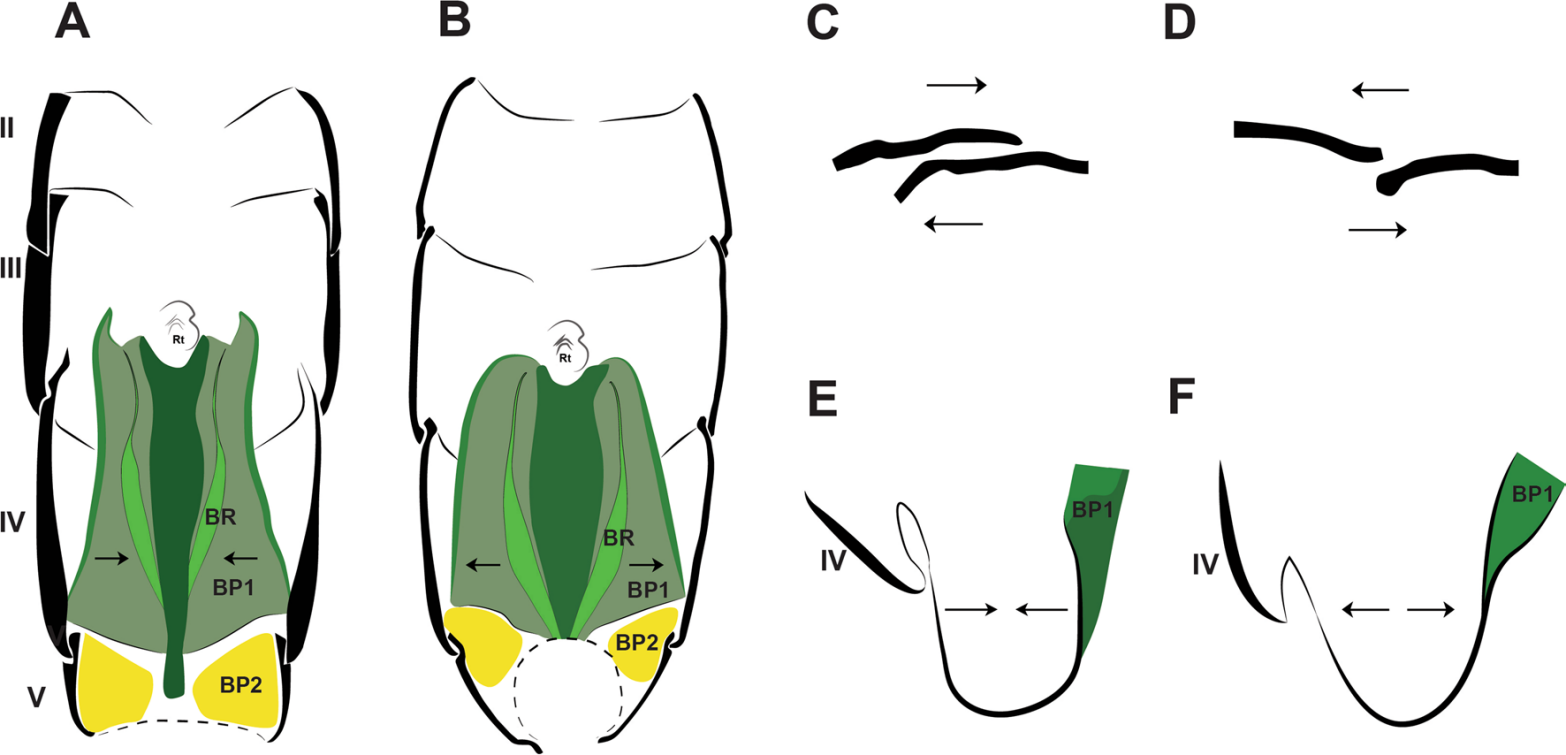
Schematic image of the functioning of cuticular structures such as tergites, sternites, basal plates, and sclerites during jumping behavior in *Orchesella cincta*. **A** Furca extended. **B** Furca flexed. **C** Tergites of the 3rd and 4th abdominal segments (furca extended). **D** Tergites of the 3rd and 4th abdominal segments (furca flexed). **E** Transversal view of basal plate 1 (Furca extended). **F** Transversal view of basal plate 1 (furca flexed). BP1: basal plate 1, BP2: basal plate 2; Rt: retinaculum; BR: basal rods

**Fig. 7:**
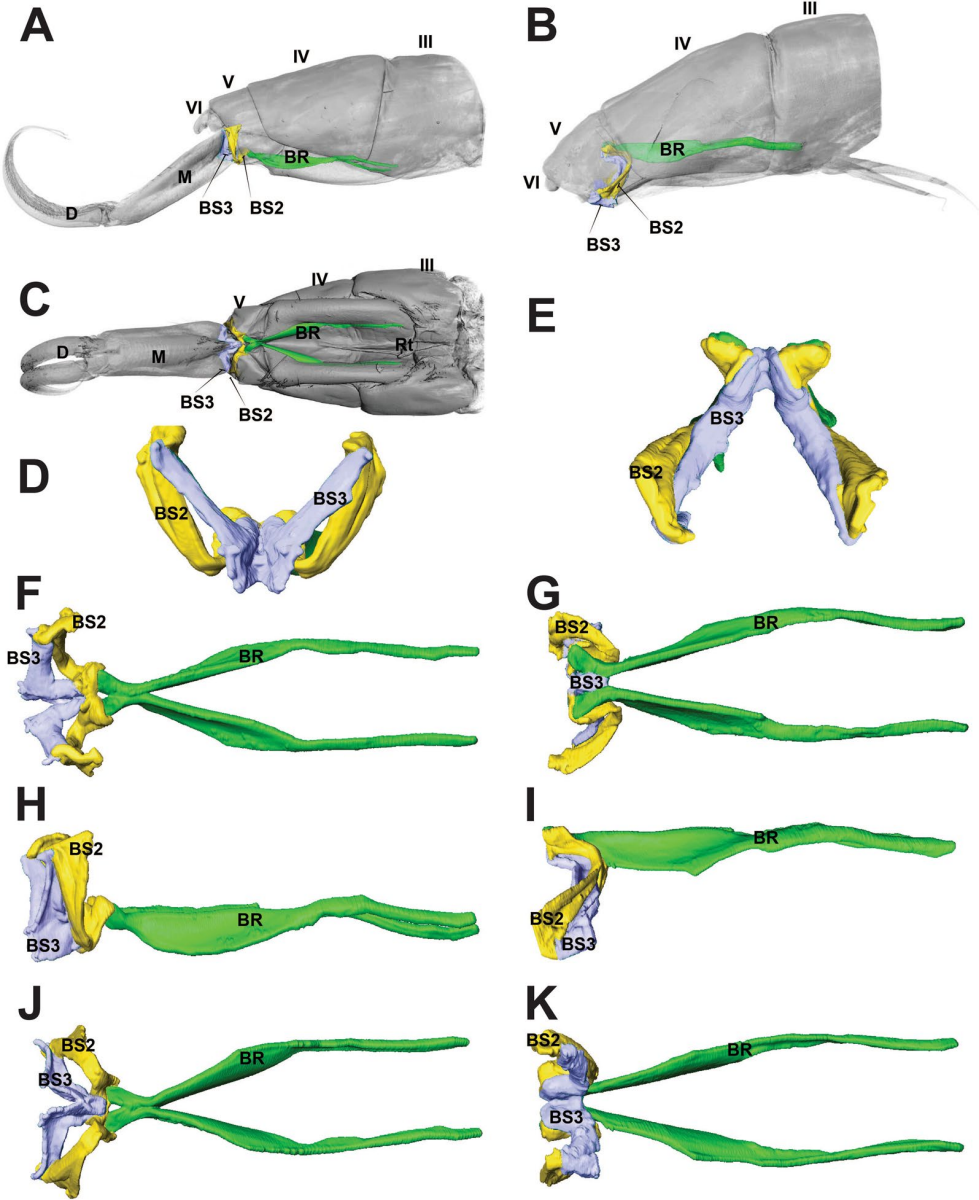
Comparative view of the basal sclerites when furca flexed and furca extended in *Orchesella cincta*. **A** Lateral view of the jumping apparatus (furca extended). **B** Lateral view of the jumping apparatus (furca flexed). **C** Ventral view of the jumping apparatus (furca extended). **D**–**K** Basal sclerites (cuticle transparency). **D** Posterior view (furca extended). **E** Posterior view (furca flexed). **F** Dorsal view (furca extended). **G** Dorsal view (furca flexed). **H** Lateral view (furca extended). **I** Lateral view (furca flexed). **J** Ventral view (furca extended). **K** Ventral view (furca flexed). BR: basal rods; BS2: basal sclerite 2; BS3: basal sclerite 3; M: manubrium; D: dens

**Fig. 8:**
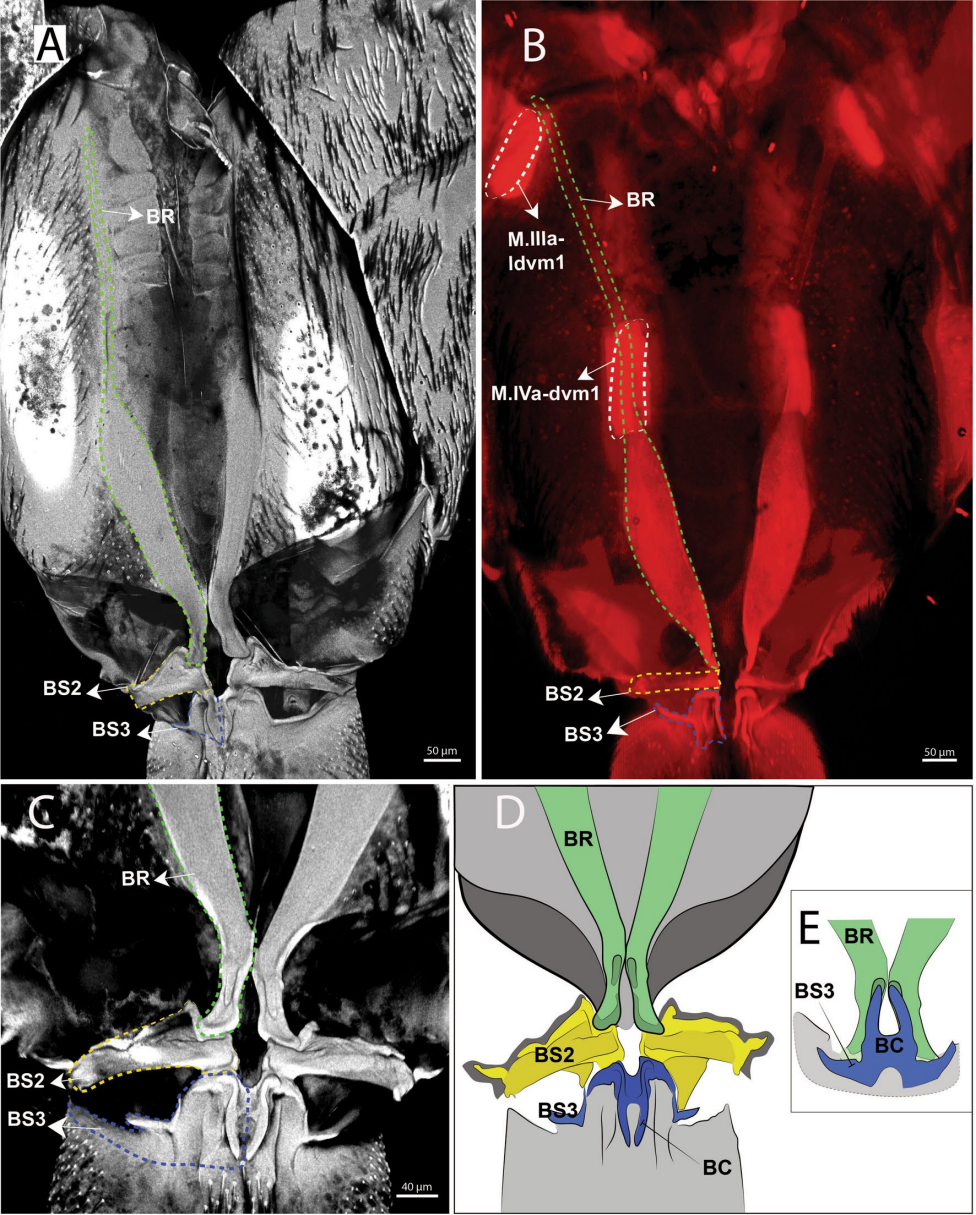
cLSM images showing the muscles which connect to BP1, the sclerites with which the furca articulates with the abdomen and the basal condyle in *Orchesella cincta*. **A** Vental view of the basal sclerites at 405 nm. **B** Ventral view of the basal sclerites at 555 nm (stained with phalloidin). **C** Ventral view of the basal sclerites at 405 nm. **D**-**E** Schematic images of the functioning of the abdominal basal sclerites in the extended (**D**) and flexed (**E**) furca states. BR: basal rods; BS2: basal sclerites 2; BS3: basal sclerites 3; BC: basal condyle

BP1 is the most prominent basal plate, originating from the inner posterior border of the 3rd abdominal segment, medially close to the retinaculum. Posteriorly it forms a border with BP2. BP1 has the most complex architecture. It is shaped like a swim float and has 2 walls, one on the inner side and another on the lateral border, allowing the board to assume a folded shape when seen in transversal or frontal perspective (Figs. 5A–F, 6A, B). The basal rod (BR) is the basal sclerite of BP1. It begins anteriorly, almost at the edge of the 3rd abdominal segment, on the side of the inner wall. The BR is initially narrow, assuming a thicker and flatter surface from the middle of BP1 up until the posterior end, where it connects to BP2 (Figs. 6A, B, 7A–K, 8A, B). At the posterior end of the basal rod are two finger-shaped depressions into which the basal condyle (bc) fits in the flexed furca state (Fig. 8C, D, E).

**Fig. 9:**
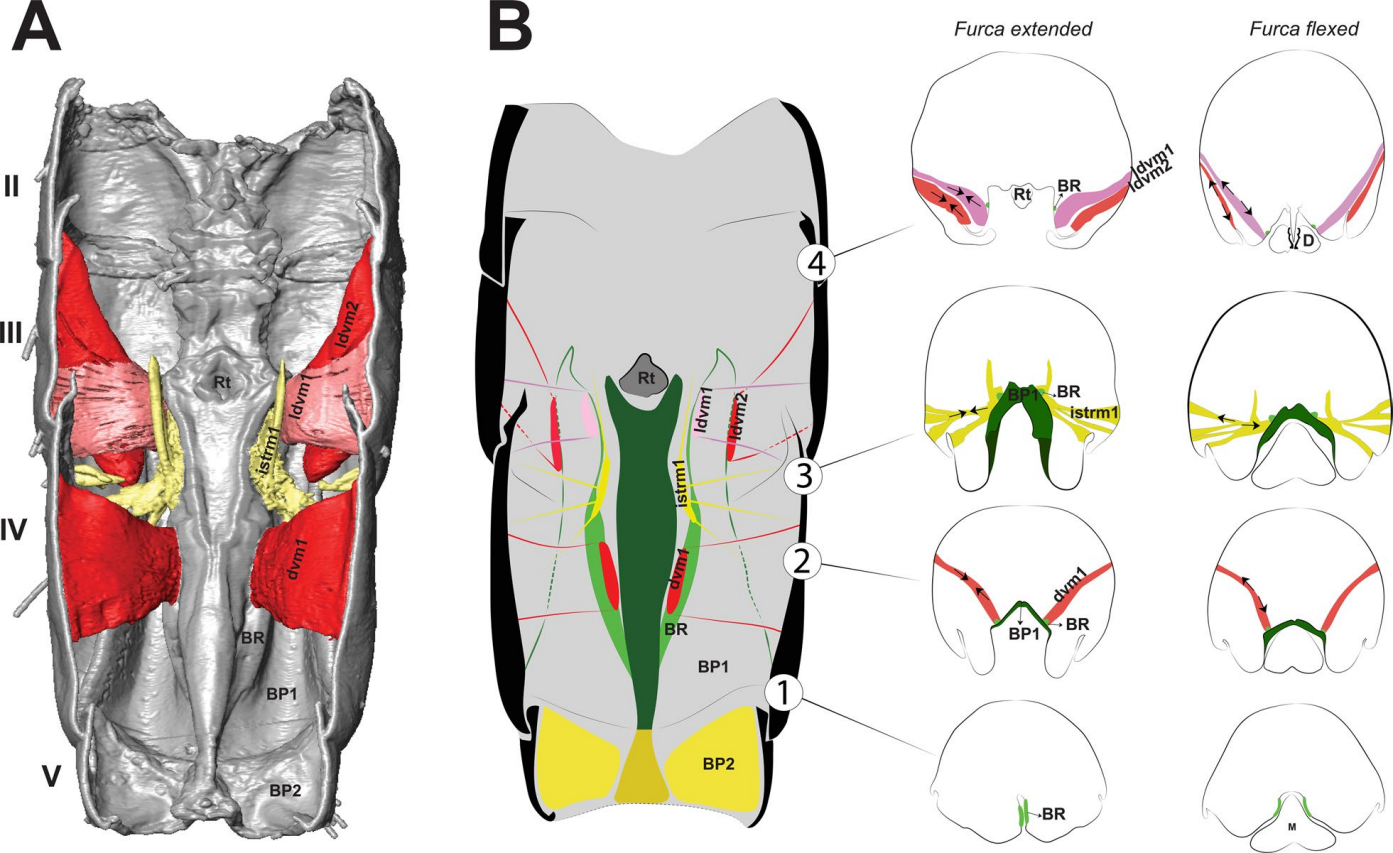
The musculature connected to BP1 in *Orchesella cincta*. Muscles M.IVa-dvm1, M.IIIa-ldvm1, M.IIIa-ldvm2 and M.IIIa-istrm1 (reconstruction of IIa-Va, seen from dorsal). **A** Muscles M.IVa-dvm1, M.IIIa-ldvm1, M.IIIa-ldvm2 and M.IIIa-istrm1. **B** Comparative schematic view of the functioning of the muscles connected to BP1 with furca extended and flexed. (1) Posterior region of the 4th abdominal segment. (2) Middle of the 4th abdominal segment. (3) Anterior region of the 4th abdominal segment. (4) Middle region of the 3rd abdominal segment. BP1: basal plate 1; BP2: basal plate 2; BR: basal rods; Rt: retinaculum; M: manubrium

**Fig. 10:**
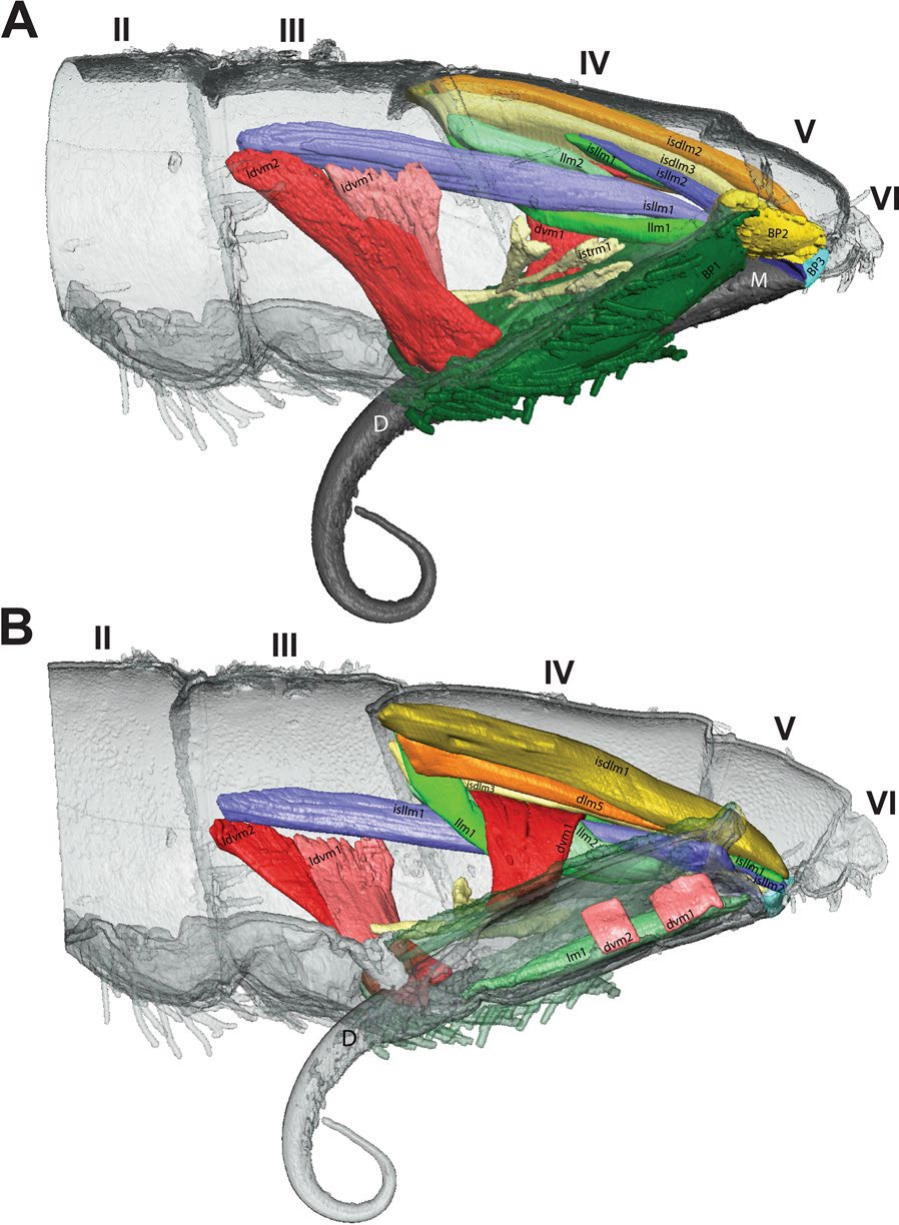
MicroCT morphological reconstruction of the lateral of the 2nd–6th abdominal segments, showing the muscles attached to BP1, BP2 and BP3 in flexed furca state. **A** External lateral side view of the jumping apparatus. **B** Internal lateral side view of the jumping apparatus. BP1: basal plate 1; BP2: basal plate 2; BR: basal rods; M: manubrium; D: dens

The muscles working anteriorly on BP1 and BR are M.IIIa-ldvm1 and M.IIIa-ldvm2, which connect the internal and external lateral wall of BP1, respectively with the lateral region of the 3rd abdominal segment. M.IIIa-istrm1, originating medially at the inner wall of BP1, connects anteriorly to the muscular center of the retinaculum, laterally to abdominal segments III and IV, as well as posteriorly to the lateral wall of BP1. In the middle of BP1, connected to the internal lateral wall, M.IVa-dvm1, the main dorsoventral muscle, can be found, responsible for deforming BP1 and BR (Figs. 3A, B, 4, 8B, 9A, B, 10A, B).

### Basal plate 2 (BP2) and basal sclerite 2 (BS2)

**Fig. 11:**
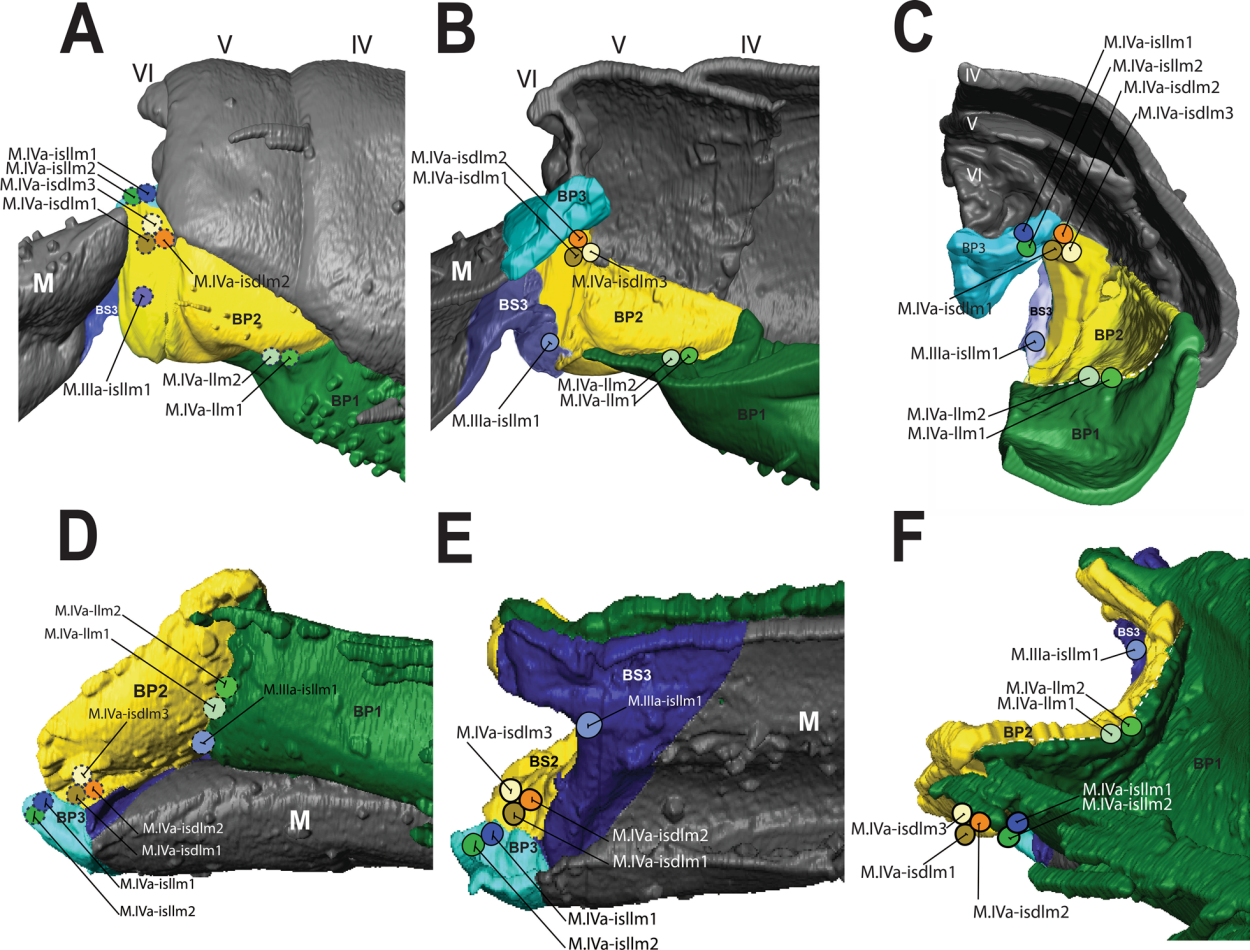
Morphological reconstruction of the lateral side of the 4th–6th abdominal segments, BP1, BP2 and BP3, the basal sclerites in *Orchesella cincta* and the insertion points of the musculature. **A**–**C** Furca extended; **A** External lateral view. **B** Internal lateral view. **C** Internal frontal view. **D**–**F** Furca flexed; **D** External lateral view; **E** Internal lateral view; **F** Internal frontal view. Dashed white line showing the border between BP1 and BP2 where there is strong deformation when the furca is flexed. BP1: basal plate 1; BP2: basal plate 2; BP3: basal plate 3; BS3: basal sclerite 3; M: manubrium

BP2 is triangular in shape and is positioned ventrolaterally, forming a corner between the abdomen and the furca. In the flexed furca state, this plate is telescoped internally. BS2 originates ventrally in BP2 (between BP1 and BP3) and extends vertically and laterally along the posterior edge of BP2, connecting to the ventral lateral edge of tergite V at the border between the abdomen and the furca (Figs. 5A–F, 7A–K, 8A–C, 11A–F).

Five muscles connect to BP2, two of them—M.IVa-llm1 and M.IVa-llm2—to the anterior ventrolateral portion (on the border between BP1 and BP2). These two muscles run laterolongitudinally together, assume a spiral shape, cross and support the muscle M.IVa-dvm1 and connect dorsally at the border between the 3rd and 4th abdominal segments. Laterally, on the dorsal surface at the corner of BP2, the muscles M.IVa-isdlm1, M.IVa-isdlm2 and M.IVa-isdlm3 connect (i.e. to the basal plate) having the origin connection point dorsally on the border between the 3rd and 4th abdominal segments (Figs. 3A, B, 4, 10A, B, 11A–F).

### The basal plate 3 (BP3) and basal sclerite 3 (BS3)

**Fig. 12:**
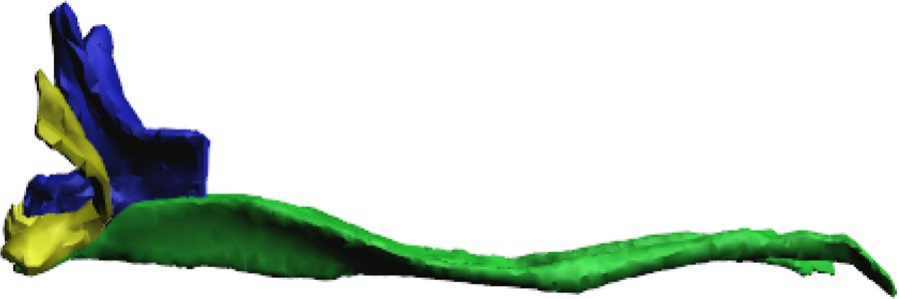
Interactive three-dimensional model of the movement of basal sclerites BR, BS2 and BS3 between the flexed and extended furca phases. In the online version, this content can be accessed from the link available in Supplementary Information as Additional file 1 (10.1186/s12983-022-00463-y.). After downloading open the file in Adobe Reader and to activate the animation, click on the figure and rotate the object using the mouse

At the base of the furca, is the basal plate 3 (BP3), the point of articulation between the basal sclerites BS2 and BS3. Here I consider as BS3 the anterior part of the furca that articulates with the abdomen, and in flexion it pushes the basal sclerites to the center of the body. This sclerite connects to BP3 laterally and then extends ventrally in a V-shape (in transverse view) and merges in the midline of the manubrium. At the base of the BS3 there is a finger-shaped basal condyle that forms the point of contact between the furca and BR when the furca is flexed (Figs. 5A–F, 7A–K, 8A–E, 11A–F, 12 (Additional file 1), 13A–D).

**Fig. 13:**
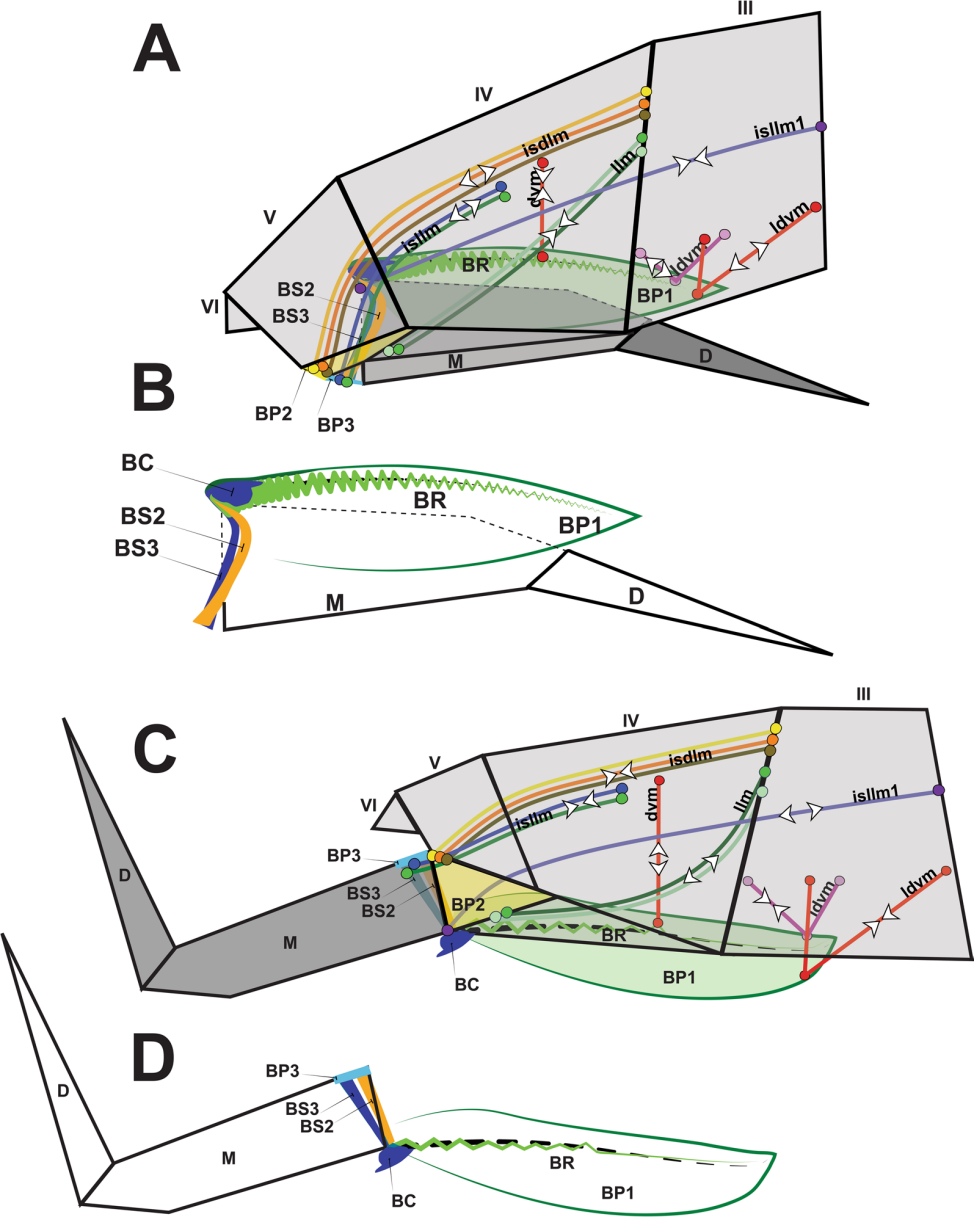
Schematic representation showing how basal plates BP1, BP2 and BP3 and the muscles function in the construction of the spring mechanism. **A**, **B** Furca flexed. **C**, **D** Furca extended. M: manubrium; D: dens; BR: basal rods; BC: basal condyle; BS2: basal sclerite 2; BS3: basal sclerite 3; BP1: basal plate 1; BP2: basal plate 2; BP3: basal plate 3

**Fig. 14:**
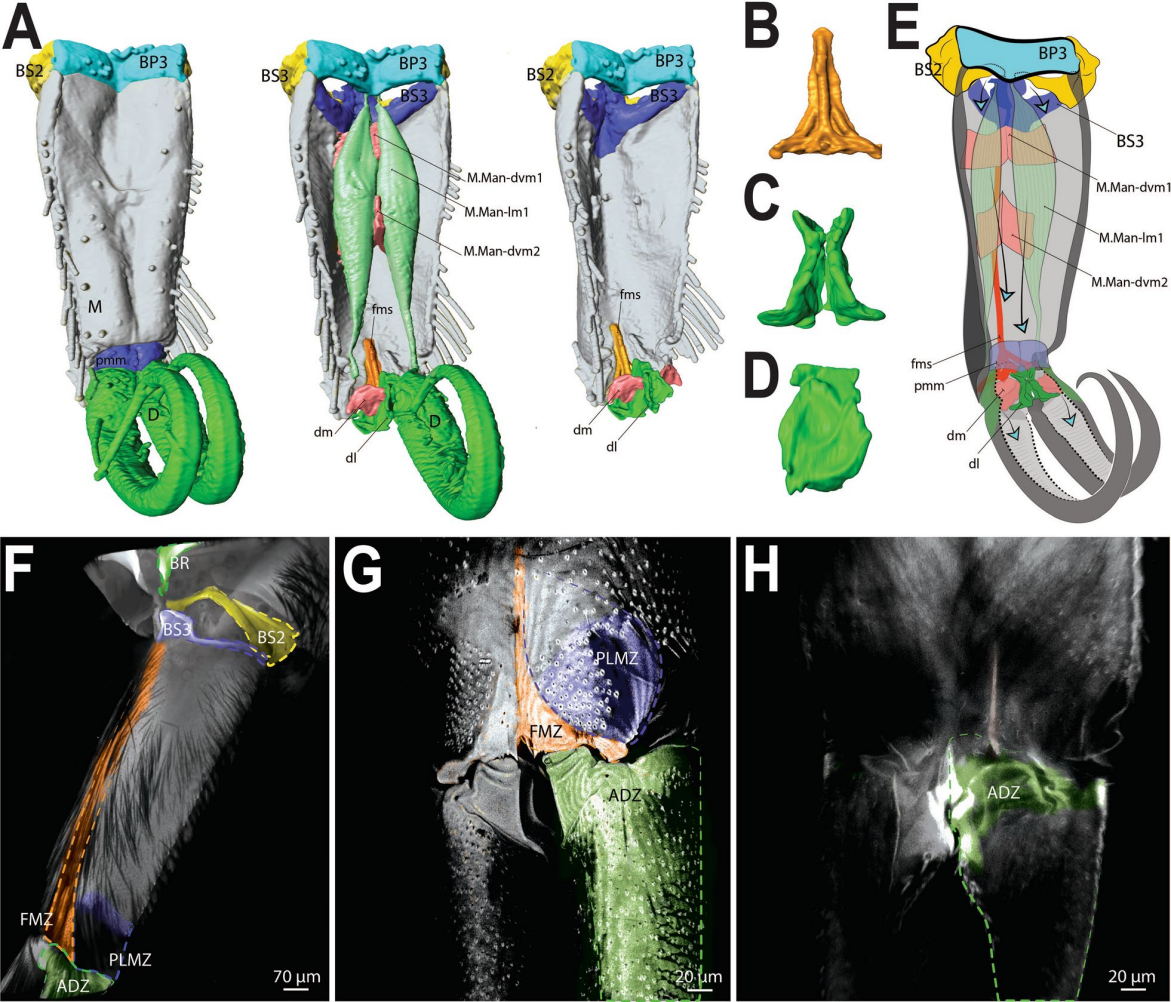
Morphological reconstruction of the furca and a propose for the potential energy storage zones. **A** Structures of the furca; **B** T-shaped furcular sclerite of the manubrium (fms); **C** Dens lock; **D** One side of the dens lock (seen from the inside); **E** Schematic representation showing structures of the furca; The blue arrows are illustrating the hemolymph flow at the moment of the furca extension. **F**–**H** cLSM images (405 nm) showing through cuticle autofluorescence potential energy storage zonesof the jumping mechanism. M: manubrium; D: dens; BR: basal rod; BS2: basal sclerite 2; BS3: basal sclerite 3; BP1: basal plate 1; BP2: basal plate 2; BP3: basal plate 3; BR: basal rods; fms: furcular manubrium sclerite; dm: membrane of dens; dl: dens lock; pmm: posterior membrane of manubrium; FMZ: furcular manubrial zone; PLMZ: posterolateral manubrial zone; ADZ: anterior zone of dens

Three abdominal muscles connect to BP3 and BS3, they are: M.IIIa-isllm1, M.IVa-isllm1,﻿ M.IVa-isllm2.  M.IIIa-isllm1, starts dorsolaterally in the 3rd abdominal segment and extends posteriorly to connect at the base of the furca, in the BS3 (Figs. 3A, B, 4, 10A–B). M.IVa-isllm1 and﻿ M.IVa-isllm2 connect on the dorsal lateral part of BP3 and originate laterally, in the middle tergite of the 4th abdominal segment. Medially at the BP3 originate the M.Man-lm1, which connects posteriorly at the base of the posterior manubrium membrane (pmm) (Figs. 3A, B, 4, 10A, B, 14A–H, 17A, B).

### The furca: manubrium and dens

The furca connects ventrally to the 4th abdominal segment through BP3 and BS3, and when flexed it extends anteriorly along the midline to the first abdominal segment. This medial appendix is made up of 2 main parts, anteriorly the manubrium and posteriorly, the dens (Figs. 2A–C, 3A, B, 4, 5A, B, 10A, B, 14A–H). With a tubular shape the manubrium is fused medially, and in the posterior portion there are two structures, dorsally a T-shaped furcular sclerite of the manubrium (fms), and ventrally a flexible membrane (pmm), both elastic structures border the dens. Anteriorly the dens is formed by two pads that together form the dens lock (dl), and then bifurcate posteriorly, and with a series of crenulations assume curved shape. These structures have internal channels through which the hemolymph flows between furca and abdomen, in both directions (Fig. 14E). Internally the dens pad, there is a transverse membrane of dens (dm). At the base of the dens, internally between each of its parts, there are a series of locks, where the retinaculum connects when the furca is flexed (Figs. 16F, G, 17A).

There are three pairs of muscles in the furca, present exclusively in the manubrium. M.Man-lm1 is a longitudinal muscle that originates medially in the anterior portion of BP3 and connects posteriorly on the lateral side of the manubrium, right at the base of pmm, close to the articulation point with the dens. The other two pairs of muscles are dorsoventral, M.Man-dvm1 is present anteriorly, and M.Man-dvm2 solely in the middle portion of the manubrium (Figs. 3A, B, 14A, E).

### The retinaculum

Ventromedially in the posterior portion of the 3rd abdominal segment, the retinaculum is situated. This very small and unremarkable structure has an elastic cuticular composition that permits deformation, and an architecture capable of holding the furca at rest. It is a triangular-shaped structure with 2 arms at its end (the rami), each one with 4 teeth. At the base of each of the rami, a pivot point is laterally located (Figs. 2A–C, 16A–E).

**Fig. 15:**
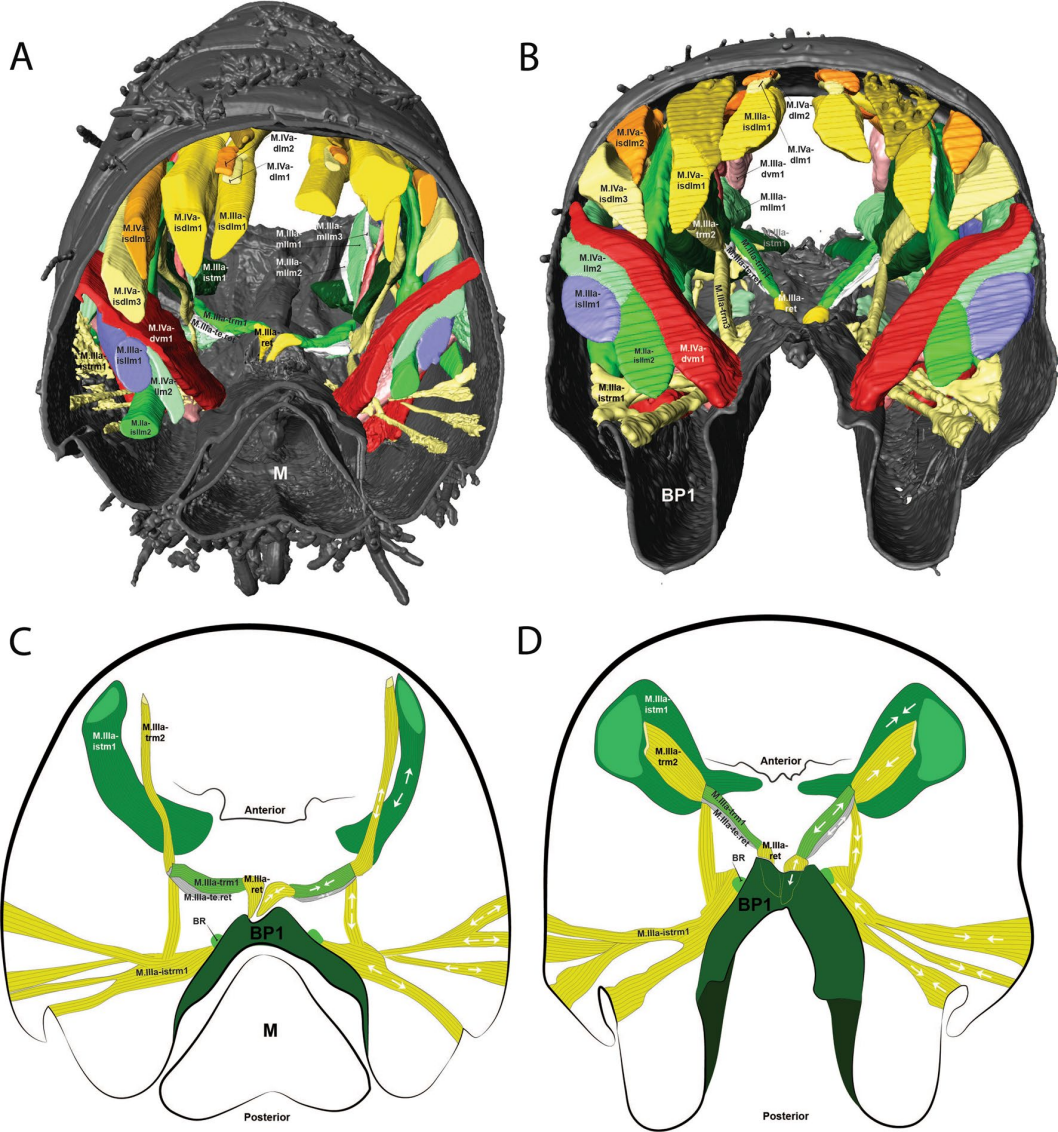
Comparative transversal view of the 4th abdominal segment in *Orchesella cincta* reconstructed with MicroCT. **A** Furca flexed; **B** furca extended; **C**, **D** schematic representation of the functioning of the jumping apparatus. **C** Furca flexed; **D** furca extended. M: manubrium; BP1: basal plate 1; BR: basal rods

Internally a complex muscular system supports the inherent movements of this structure. Connecting internally to the retinaculum, on the upper inner portion of each rami, are the short M.IIIa-ret muscles. At the base of this is a point of connection with M.IIIa-trm1 and the M.IIIa-te.ret tendon. Both these muscles connect internally to a complex muscle center composed of M.IIIa-trm2, M.IIIa-istrm1 and M.IIIa-istm1 (Figs. 3A, B, 4, 15A–D, 17A, B, 18A, B).

## Morphofunctional aspects related to jumping

### Spring mechanism, triggers and energy storage

The cLSM images (through cuticle autofluorescence) revealed the areas of the jump apparatus that are most elastic and have energy storage potential, here proposed as potential energy storage zones  (Figs. 8A, C, 14F–H). The spring mechanism is generated in BP1 (BR), BP2 (BS2), BP3 (BS3), mainly from the deformation of their respective basal sclerites (Fig. 11D–F). Such deformation and energy storage is made possible by the elasticity of these structures, potentially rich in resilin, as shown by Büsse & Gorb [20] studying the mouthpart cuticle in damselfly larva (Odonata). The tension in the sclerites is generated by muscular action, hydrostatic pressure and in large part by tension between the furca and the basal sclerites when furca is flexed (Fig. 12). In addition to BP1, BP2 and BP3, in the furca three main potential energy storage zones can be identified: the posterior furcular manubrial zone (FMZ) with strong posterior furcular manubrium sclerite (fms), and laterally the posterolateral manubrial zone (PLMZ) where a flexible posterior manubrium membrane  (pmm) is present. Both posterior manubrial zones, articulate with the anterior zone of dens (ADZ), the main point of contact between the furca and the ground surface (Figs. 7A–K, 8A–E, 12, 14F–H).

The spring mechanism is intensified by the presence of the trigger, the retinaculum, which increase the tension between the basal plates and prevents the basal sclerites from returning to a relaxed state. At the base of the furca a basal condyle (bc) at BS3 intensifies the tension on the basal sclerites and extends posteriorly along with the manubrium as a keel, coming into direct contact with the ventral part of the abdomen when the furca is flexed (Figs. 10A, B, 11A–F, 12, 13A–D).

### Hydrostatic pressure

Hydrostatic pressure plays a passive role in jumping by increasing the tension of the basal plates, and an active role by increasing the efficiency of the jump in coordination with the muscles. Thereby the hemolymph is concentrated inside the body cavity (and also in the furca), tensing its walls in a dorso-ventral and longitudinal movement when the furca is flexed. With the release of the furca, this hydrostatic volume is directed ventrally and posteriorly toward the furcular cavity (Figs. 14E, 17A, B).

In the abdomen, the muscles related to the increase in hemolymphatic pressure are mainly the dorso-longitudinal muscles (M.IIa-isdlm1, M.IIIa-isdlm1, and the dlm), dorsoventral (M.IVa-dvm1), ventral and lateral longitudinal muscles. The contraction of these muscles causes a reduction in the space between the abdominal segments and, consequently, an increase in hydrostatic pressure. At the moment of extension, M.IIIa-istm1 may have an important role in directing pressure to the basal plates by deforming the 3rd abdominal segment dorsoventrally and decreasing the opening for hydrostatic flow through the posterior abdominal region. The pleural muscles M.IIIa-istrm1, M.IIIa-ldvm1 and M.IIIa-ldvm2 and the ventral longitudinal lateral intersegmental muscles MIIa-isllm1, MIIa-isllm2, MIIa-isllm3, MIIa-isllm4 and M.IIa-isllm5 could act by regulating the hydrostatic pressure between the lateral tergites (IIIa-IVa), sternite III and BP1 in the anteroposterior flow towards the opening cavity of the furca (Figs. 3A, B, 4, 9A, B, 10A, B, 11A–F, 13A–D, 14E, 17A, B).

**Fig. 16:**
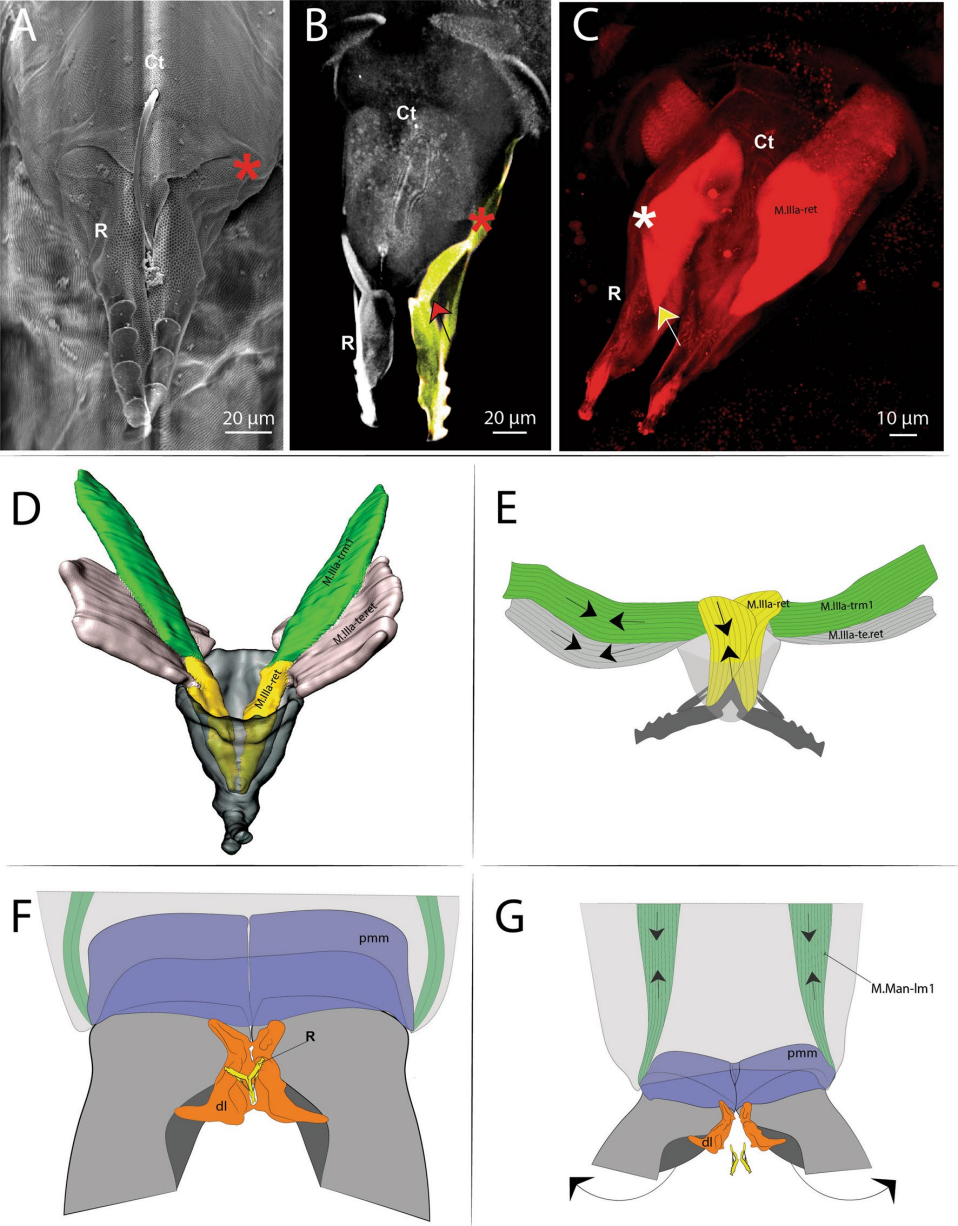
Morphology and function of the retinaculum. **A** Scanning electron microscopy (SEM) image (anterior view) of the retinaculum; **B** Confocal microscopy (cLSM) image at 405 nm (view from anterior) of the retinaculum; yellow marking showing the Rami; red arrow showing the point of connection of the M.IIIa-ret muscle internally to the rami; **C** Confocal microscopy (cLSM) image at 555 nm (stained with phalloidin) (view from anterior) of the retinaculum; yellow arrow showing the point of connection of the M.IIIa-ret muscle internally to the rami; **D** Morphological reconstruction using micro computer tomography (MicroCT) (view from posterior) of the retinaculum; **E** Schematic representation showing the muscular function of the retinaculum; **F**, **G** Schematic representation showing a hypothesis on how the furca may be released from the retinaculum. Ct: corpus tenaculi; R: retinaculum ramus or rami; DL: dens lock; The “*” means the Pivot point of articulation between Rami and Corpus tenaculi

**Fig. 17:**
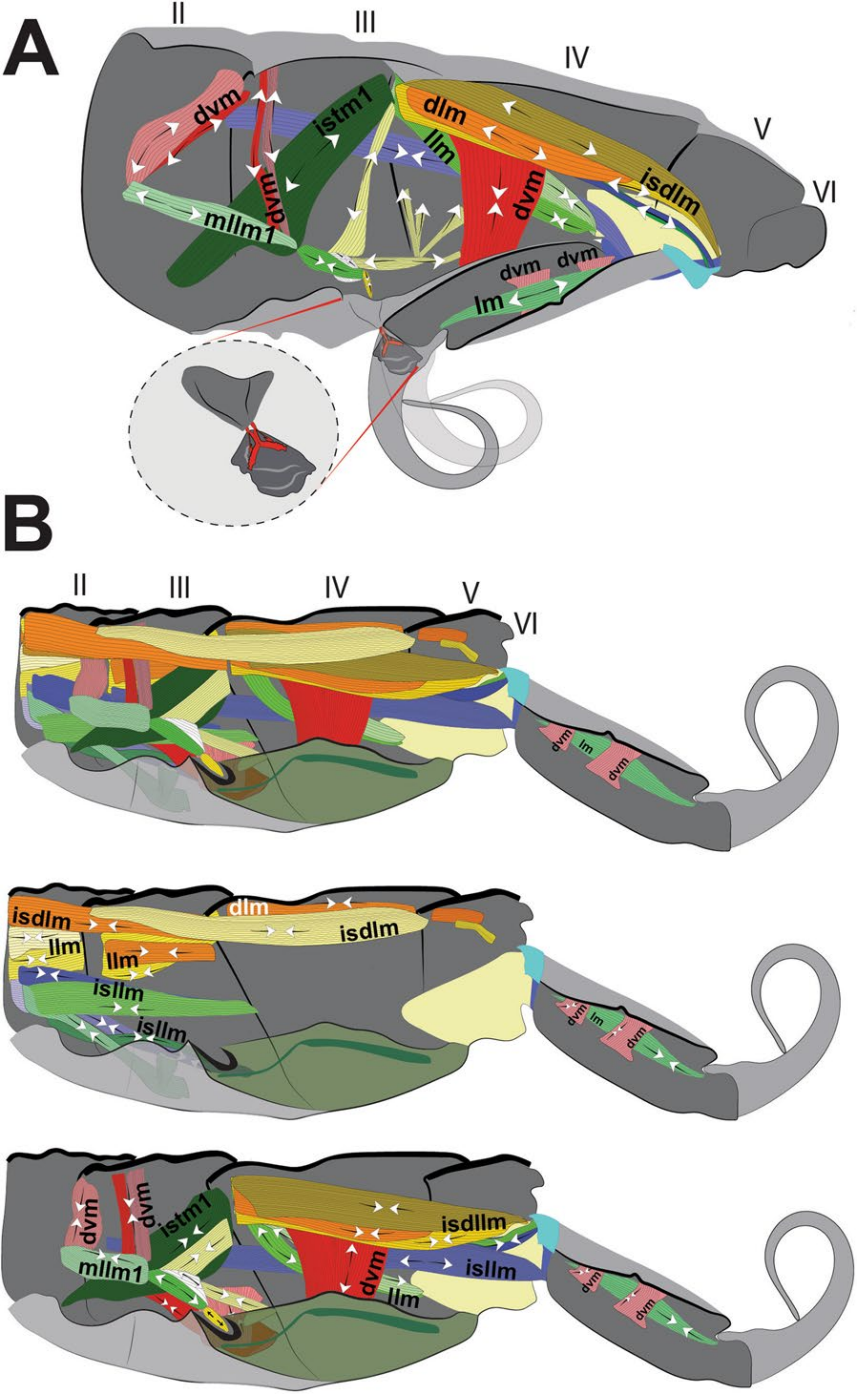
Schematic view of the functioning of the jumping apparatus in *Orchesella cincta*. **A** Internal side view (flexed furca); the dashed line forming a circle shows the retinaculum attached to the dens lock. **B** Internal side view (extended furca). The arrows show the direction of the muscle fibers.* Dvm* dorsoventral muscles;* llm* lateral longitudinal muscles;* isllm* intersegmental laterolongitudinal muscles;* isdlm* intersegmental dorsolongitudinal muscles;* dlm* dorsal longitudinal muscles

**Fig. 18:**
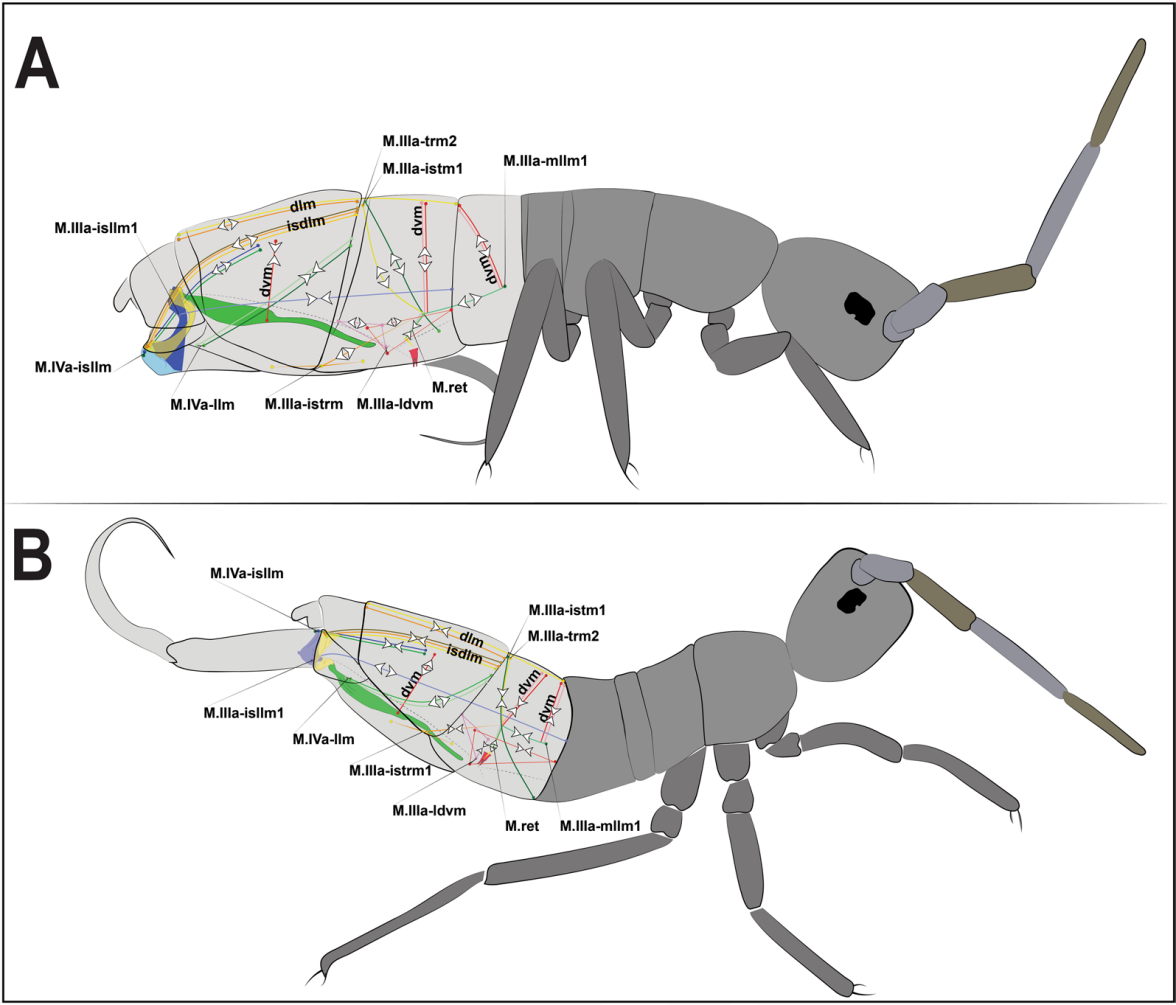
Schematic representation of the jumping apparatus in the flexed furca (**A**) and extended furca (**B**) phases in *Orchesella cincta.* Dvm: dorsoventral muscles; llm: lateral longitudinal muscles; isllm: intersegmental laterolongitudinal muscles; isdlm: intersegmental dorsolongitudinal muscles; dlm: dorsal longitudinal muscles

In the furca, the dorsoventral (M.Man-dvm1, M.Man-dvm2) and longitudinal muscles (M.Man-lm1) are involved in controlling hemolymphatic pressure by compressing the cuticle walls, effectively injecting hemolymph into the dens cavity. As already mentioned, the muscles (M.Man-lm1) potentially also act by releasing the furca from the retinaculum (Figs. 16F, G, 17A, B, 18A, B).

No experiments were performed to measure hemolymphatic pressure and test this hypothesis proposed by Manton [10]. Such an interpretation comes from the comparative analysis in this study, between specimens with extended and flexed furca. Based on the shape of the body, such as muscles and especially the overlapping between the segments, this hypothesis was reinforced (Fig. 6A–F).

### Transition between "flexed furca" and "extended furca” phases

The flexed furca phase starts with the engagement of the retinaculum in the locks of the dens (dl), with the help of muscles M.IIIa-trm1, M.IIIa-te.ret and M.IIIa-ret, which through their contraction set up the hook, when the furca returns to the ventral side of the abdomen (Figs. 16D, E, 17A, B). The return of the furca to the abdomen potentially occurs by the combined contraction of muscles M.IVa-dvm1 (which connects to BP1), M.IVa-llm1 and M.IVa-llm2 (which connect at the border between BP1 and BP2) and the long M.IIIa-isllm1 (which connects to BS3 at the base of the furca) (Figs. 3A, B, 10A, B, 11A–F, 13A–D, 17A, B, 18A, B). The furca extension phase begins with the release of the furca by the retinaculum when extension is needed. It has been proposed by Manton [10] that the furca is released from the retinaculum by hydrostatic pressure alone (this will be addressed in the discussion section). Here I propose an alternative hypothesis, that the contraction of the longitudinal manubrial muscle M.Man-lm1 through the articulation with the dens (which is almost like a knee), creates the pull on the posterior side of manubrium, the posterior manubrial membrane (pmm) and their sclerites. This causes an opening of the dens pads, resulting in the opening of the dens lock, and the release of the furca (Figs. 16F, G). Subsequently, the contraction of muscles M.IVa-isdlm1, M.IVa-isdlm2, M.IVa-isdlm3, M.IVa-isllm1 and M.IVa-isllm2 could extend the furca (Figs. 10A, B, 13A–D, 17A, B, 18A, B) and, together with the spring and the hemolymphatic pressure mechanisms (or even without, but with loss of efficiency), result in jumping.

## Discussion

It has been suggested [8, 9, 13, 14] that the strategies and mechanisms involved in jumping vary between species that have distinct segmentation and are cylindrical in shape (such as *Orchesella cincta* and *Tomocerus* spp.), and those with fused segmentation which are globular in shape (such as *Sminthurus* spp.). However, all taxa (except for those with secondary loss of the furca and retinaculum) essentially and primarily use the furca as a catapult and the retinaculum as a trigger [10, 12–14] . Favret et al. [21] additionally suggested that the ventral tube, when adhered to the substrate, could serve to act on the direction and trajectory of the jump, but this is not something evaluated in this study.

Several studies [10, 12–14] agree that the basal plates play an important role in creating the spring mechanism. Manton [10] and Christian [13] recognized the basal rods as the structures in which elastic energy is stored. Eisenbeis and Ulmer [12] and Brackenbury and Hunt [14] suggested that this energy is stored not only in the basal rods, but also in other basal sclerites (such as BS2 and BS3). Eisenbeis and Ulmer [12] were the first to suggest that elastic energy could be stored by the resilin present in the cuticle of the basal plates and especially in the basal sclerites of springtails. Gronemberg [2] declared the energy storage mechanism in springtails as occurring through the cuticle, without mentioning the resilin or the basal sclerites. According to Sudo et al. [9], the elastic energy produced is stored in the muscles and subsequently released as kinetic energy, but there is no mention of which muscles specifically. I propose that the energy is stored in BP1, BP2 and BP3 and their respective sclerites (BR, BS2 and BS3), and also in the potential energy storage zones  demarcated in the furca (Figs. 14F–H). Evidence for this can be found in the cLSM images provided by this study, which at a wavelength of 405 nm suggest the presence of resilin in these structures, corroborating Burrows et al. [1] and Büsse and Gorb [20].

There is a converging opinion, and I share it, that hydraulic pressure acts on the efficiency of the jump [10, 12–14, 22]. Manton [10] actually hypothesized that furca extension and jumping are not brought about directly by muscle force, but predominantly by hydraulic pressure. However, this was disputed by Christian [13] and Eisenbeis and Ulmer [12] (and also this study), who put forward the hypothesis that furca extension is primarily the result of muscular force, independent of hydrostatic pressure. On the other hand, Brackenbury and Hunt [14] rejected the idea of furca extension being brought about predominantly by muscular action, describing the muscles attached to the manubrium as having a very weak line of action and a small total mass. In my opinion, muscular force would be sufficient to open the furca and even to permit jumping, but would be incomparably less effective alone than when combined with hydrostatic force. Eisenbeis and Ulmer [12] show and describe possible channels through which hemolymph flows. Cylindrical and segmented species like *O. cincta* possess the ability to telescope the abdominal segments and the tergo-pleural arches [10, 13], primarily by using of the dorsal and lateral longitudinal muscles of the trunk [10]. This phenomenon has been suggested to be the main mechanism behind hydraulic pressure [10, 14], and by my observations support this hypothesis.

It is widely accepted that the retinaculum acts as a trigger, and by holding the furca permits the creation of the spring mechanism [8–10, 12–14]. According to Manton [10], the distension of the retinaculum is the result of the hydrostatic pressure generated by the trunk muscles, while its retraction is mediated by the action of the muscles directly connected to it (M.IIIa-ret). However, it is still not clear how the retinaculum could be selectively everted—in other words why such hydraulic pressure does not act at the same time, for example, in the eversion of the ventral tube. Manton [10] suggested that the pleural muscles M.IIIa-istrm1, M.IIIa-ldvm1 and M.IIIa-ldvm2 may be directly involved in bracing the flexible basal parts of the body wall against increased hydrostatic pressure and thus strengthening the action of the longitudinal sternal muscles (M.IIa-isllm3, M.IIa-isllm4 and M.IIa-isllm5) in the vicinity of the retinaculum. My observations make it seem much more likely that hydrostatic pressure is not necessary for the release of the furca by the retinaculum, and that this occurs by the exclusive force of one muscle, M.Man-lm1.

Despite the differences in the jumping mechanisms among Collembola species, jumping in all of them features the following phases: (1) the take-off phase, between the start of furca movement and the moment the animal stops touching the ground; (2) the aerial phase, which ends when the animal touches the ground again; and (3) the landing phase, when the animal touches the ground for the first time after the jump [9, 13]. Christian [13] described jumping in *O. cincta* as involving similar movements to jumping in *Heteromurus nitidus* (Templeton, 1835), a closely phylogenetically related Entomobryid, though *O. cincta* did not jump as far or as high. Jumping in *O. cincta* starts with a change in the longitudinal axis of the body. The animal bends, bringing head, legs and furca toward the ground. With the release of the furca, there is a projection of the body backward. The body acquires angular impulse, then moves over the dens until the animal is standing up straight and aligned with the longitudinal axis of the body. The weight and force of the body is then transferred to the distal part of the dens and the substrate surface, which relieves tension in the manubrium-dens joint and confers stability to the movement. For the extension of the furca the body assumes a curved concave dorsal contour, probably due to the contraction of direct and indirect extensor muscles [13]. Eisenbeis and Ulmer [12] recognize as extensors of the furca the muscles M.IVa-isdlm1, M.IVa-isdlm2 and M.IVa-isdlm3, together with the longitudinal muscles. According to them, the extensor traction acts first on the medial and lateral parts of BP3 and BS3, and is then transferred to the manubrium, with opening aided by increased hemolymphatic pressure. Finally the manubrial muscles M.Man-dvm1, M.Man-dvm2 and M.Man-lm1 expand and spread the dens and compress the manubrium [12]. In Christian’s [13] experiments, *O. cincta* had a maximum extension speed equivalent to 290 rad s^−1^, which indicates how quickly the manubrium is unfolded in this species. The angle of body rotation at take-off was 71°, with a rotation speed of 102 rad s^−1^ after the take-off phase. The animals achieved a height of 6 mm during the jump. Apparently, all springtails exhibit a backward rotation, as shown by Christian [13] and Sudo et al [8, 9]. Brackenbury and Hunt [14] hypothesized that this is explained by the position and length of the the furca, which ends below or in front of the body's center of mass. The release of the furca thus causes an imbalance to this center, and the furca creates a backward rotation. Brackenbury and Hunt [14] and Sudo et al. [8] agree that spinning while jumping could be a waste of energy, but Sudo et al. [8] also suggest that it may be a mechanism by which springtails control the height and direction of their jump. Christian [13], although he did not make cinematographic images, suggested that although the jump may seems to be random as to its direction, a pattern of falling landing in a standing posture, or getting up quickly, was observed.

## Conclusions

Jumping in springtails involves mechanisms and a morphological apparatus unique to this group of arthropods. By studying the morphology of a springtail and comparing the "furca flexed" and "furca extended" phases, we were able to gain a deeper understanding of the mechanisms involved in jumping. Springtails with distinct segmentation possibly have hydrostatic regulatory mechanisms that are distinct [i.e. different] from those with fused segmentation, by using the overlapping of the segments to increase hemolymphatic pressure. Energy appears to be stored in resilin elastic cuticular structures. A detailed morphological study revealed interesting cuticular characters such as basal plates and sclerites. The present study provides useful information for future phylogenetic and morphofunctional studies. To gain a better insight into the precise movements involved in the different phases of jumping, an analysis of video and image records made using a high speed camera would be helpful.

## Methods

### Specimens sampling and fixation

The morphology of the jumping apparatus was studied comparatively between 10 specimens of *Orchesella cincta* with extended (T101–T110) and 6 specimens with flexed furca (T201–T206) (Fig. 2A–C). Abdominal segments 2nd–6th were reconstructed, including cuticular structures such as basal plates and sclerites, as well as internal musculature. The animals were found in the garden of the Institute of Zoology at the Universität Rostock, in the surface layer of leaf litter, using an entomological aspirator. In an attempt to obtain springtails with a natural flexed furca (without manipulation on this structure), the specimens were placed in a freezer (-10 °C) for 15 min to have their metabolism slowed down. Then, the specimens were immersed in two fixatives depending on the microscopy procedure, Duboscq-Brasil fixative for study using Micro Computer Tomography (MicroCT), and PFA fixative (4% in PBS) for study using Confocal Laser Scanning Microscopy (cLSM). Voucher specimens are deposited at the Institute of Zoology at the Universität Rostock.

### Confocal laser scanning microscopy (cLSM)—PFA (4% in PBS)

Here, two approaches were taken: (1) to investigate the cuticular elastic structures, specimens were not stained and were exposed to a wavelength of 405 nm; (2) to investigate muscular tissues, specimens were stained with Phalloidin and exposed to a wavelength of 555 nm. For both approaches the specimens were transferred alive and immersed in PFA solution (4% in PBS) for at least 1 h. Subsequently, the specimens were washed in PBS (1 × Phosphate-Buffered Saline) solution in 3 steps of 5 min each, then washed in 0.05 NaN_3_ PBS (Natrium-Azid) solution for 5 min, and transferred to the refrigerator.

The specimens for Phalloidin staining were then subjected to another chemical treatment series. First, they were washed in PBT (PBS + Triton X-100) in 4 steps of 25 min, then, in a light-protected environment, immersed in 1 µl of phalloidin and 1 ml of PBT solution for 90 min. Subsequently they were immersed in 3 PBS baths, the first for 3 min, and the last two for 15 min, and then transferred to 1 × PBS + 0.05% NaN_3_. Finally, they were transferred to 1 × PBS + 0.05% NaN3 and refrigerated at 5 °C.

The specimens were mounted between two glass coverslips (60 mm x 24 mm), then immersed in 100% glycerin or in RapiClear 1.47, SunJin Lab (when heavily pigmented). Modeling clay was used to seal the four edges. Slides were then taken to the cLSM (LEICA Stellaris 8) and studied at 405 nm (without staining) and 555 nm (with Phalloidin staining). The slides were kept in the dark and refrigerated (5 °C) when not in use.

### Micro computer tomography (MicroCT)

The specimens were fixed for at least 1 h in Duboscq-Brasil then washed to dehydrate them in a series of different concentrations of alcohol (ethanol) starting at 20% and proceeding up to 99.8% (20%, 30%, 40%, 50%, 60%, 70%, 80%, 90% and 98.8%) (each step 10 min). They were later transferred to the Leica EMCPD300 equipment for critical point drying, then mounted with white liquid glue on toothpicks for study under Micro Computer Tomography (ZEISS Xradia 410 Versa X-Ray).

### Scanning electron microscopy (SEM)

The specimens were initially transferred to 20% ethanol and then subjected to a series of dehydrating solutions (20%, 30%, 40%, 50%, 60%, 70%, 80%, 90% and 98.8%) (each step 10 min) and critical point dried using the Leica EMCPD300 equipment. Subsequently, they were prepared on metallic pins to be sputter-coated with gold using a Sputter Coater EM SCD 004.

### 3D reconstruction and animation

Stacks of digital images obtained using MicroCT or cLSM were processed using the 3D reconstruction software Amira 2020.2. Data processing primarily involved segmentation of structures of interest—that is, the marking of specific structures at regular intervals within the image stack. On the basis of the segmentation, the software is able to create a surface rendering representing a 3D reconstruction of the morphological structure in question. The animation on Fig. 12 was performed as described by Günther et al. (2021).

### Terminology

The abdominal segments studied are II, III, IV, V, and VI. The terminology for the following abdominal structures is adopted from Eisenbeis and Ulmer [12]: BP1—basal plate 1, BP2—Basal plate 2, BP3—Basal plate 3, BR—Basal Rods. Retinaculum and furca are described from Schaller [23]: Rt—retinaculum, Ct—retinaculum körper (corpus tenaculi), R—retinaculum ramus, F—furca, M—manubrium, D—dens. I rename the basal sclerites of Basal plates 2 and 3 as BS2 (Basal sclerite 2) and BS3 (Basal sclerite 3), respectively. I propose new names to structures in the furca: BC—basal condyle, PMM—posterior manubrial membrane, FMS—furcular manubrium sclerite, DL—dens lock, DM—dens membrane, and the potential energy store zones, FMZ—furcular manubrium zone; PLMZ—posterior lateral manubrium zone, and ADZ—anterior dens zone.

The terminology for the name of the abdominal muscles is based on the following system. The names consist of three sections: Example: M.IVa-isllm1. The prefix indicates the type of structure and its previous point of origin. The prefix takes the capital letter representing the type of structure, M in this example means a muscle. Endosclerites with an E, and tendon names would begin with a T, etc. The roman numeral e in sequence represents the segment in which such a structure (M, E or T) originates. The lowercase letter directly linked to the Roman numeral indicates whether the segment is cephalic (c), thoracic (t), or abdominal (a). The stem is separated from the suffix by a hyphen. The suffix consists of an abbreviation and an Arabic numeral. The abbreviation represents the orientation and position of the muscles in the body.

## Supplementary Information

**Additional file 1. Fig 12.** Interactive three-dimensional model of the movement of basal sclerites BR, BS2 and BS3. To activate the animation, click on the figure in Adobe Reader and rotate the object using the mouse.

## Data Availability

The datasets used and/or analysed during the current study are available from the corresponding author on reasonable request. Animation content 3D present in Fig. 12 available also upon request to the author.

## Abbreviations

MLLM: Mediolateral longitudinal muscles
TRM: Transversal muscles
VTRM: Ventral transversal muscles
FMZ: Furcular manubrial zone
PLMZ: Posterolateral manubrial
ADZ: Anterior zone of dens
ISTRM: Intersegmental transversal muscles
DLM: Dorsal longitudinal muscles
DVM: Dorsoventral muscles
ISDLM: Intersegmental dorsolongitudinal muscles
ISDVM: Intersegmental dorsoventral muscles
ISLM: Intersegmental longitudinal muscles
ISLLM: Intersegmental laterolongitudinal muscles
LDVM: Lateral dorsoventral muscles
LLM: Lateral longitudinal muscles
BP1: Basal plate 1
BR: Basal rod
BP2: Basal plate 2
BP3: Basal plate 3
BS2: Basal sclerite 2
BS3: Basal sclerite 3
CT: Corpus tenaculi
RT: Retinaculum
D: Dens
M: Manubrium
F: Furca
R: Retinaculum ramus
PMM: Posterior manubrium membrane
BC: Basal condyle
DM: Dens membrane
FMS: Furcular sclerite of the manubrium
DL: Dens lock
cLSM: Confocal laser scanning microscopy
3D: Third dimension
MicroCT: Micro computer tomography
SEM: Scanning electron microscopy
PFA: Paraformaldehyde
PBS: 1X phosphate-buffered saline
PBT: PBS + Triton X-100
NaN_3_: Sodium azide

## References

[1] Burrows M, Shaw SR, Sutton GP (2008). Resilin and chitinous cuticle form a composite structure for energy storage in jumping by frog hopper insects. BMC Biol.

[2] Gronemberg W (1996). Fast actions in small animals: springs and click mechanisms. J Comp Physiol A.

[3] Ilton M, Bhamla MS, Ma X, Cox SM, Fitchett LL, Kim Y, Koh JS, Krishnamurthy D, Kuo CY, Temel FZ, Crosby AJ, Prakash M, Sutton GP, Wood RJ, Azizi E, Bergbreiter S, Patek SN (2018). The principles of cascading power limits in small, fast biological and engineered systems. Science..

[4] Eisenbeis G, Wichard W. Atlas in the biology of soil, vol. XIV; 1987. p. 437. 10.1007/978-3-642-72634-7.

[5] Hopkin SP. Biology of the springtails (Insecta: Collembola). Oxford University Press; 1997.

[6] Rusek J (1998). Biodiversity of Collembola and their functional role in the ecosystem. Biodivers Conserv.

[7] Palacios-Vargas JG, Mejía-Recamier BE. Técnicas de colecta. In: Técnicas de colecta, preservación y montaje de microartrópodos; 2007. Las Prensas de Ciencias, UNAM. p. 23–46.

[8] Sudo S, Shiono M, Kainuma T, Shirai A, Hayase T (2013). Observations on the springtail jumping organ and jumping mechanism worked by a spring. J Aero Aqua Biomech.

[9] Sudo S, Shiono M, Kainuma T, Shirai A, Hayase T (2013). The kinematics of jumping of globular springtail. J Aero Aqua biomech.

[10] Manton SM (1972). The arthropoda: habits, functional morphology and evolution.

[11] Eisenbeis G (1978). Die Thorakal- und Abdominal-Muskulatur von Arten der Springschwanz-Gattung Tomocerus (Collembola: Tomoceridae). Entomologica Germanica.

[12] Eisenbeis G, Ulmer S (1978). Zur Funktionsmorphologie des Sprung-Apparates der Springschwanze am Beispiel von Arten der Gattung Tomocerus (Collembola: Tomoceridae). Entomologia Generalis.

[13] Christian E (1979). The jump of the springtails. Naturwissenschaften..

[14] Brackenbury J, Hunt H (1993). Jumping in springtails: mechanism and dynamics. J Zool.

[15] Konopova B, Akam M (2014). The Hox genes Ultrabithorax and abdominal-A specify three different types of abdominal appendage in the springtail *Orchesella cincta* (Collembola). EvoDevo.

[16] Whalley P, Jarzembowski EA (1981). A new assessment of Rhyniella, the earliest known insect, from the Devonian of Rhynie, Scotland. Nature.

[17] Gisin H. Ökologie und Lebensgemeinschaften der Collembolen im schweizerischen Excursionsgebiet Basels. Revue Suisse de Zoologie. 1943; 50 pp.

[18] Agolin M, D’Haese CA (2009). An application of dynamic homology to morphological characters: direct optimization of setae sequences and phylogeny of the family Odontellidae (Poduromorpha, Collembola). Cladistics.

[19] Panina IV, Potapov MB, Polilov AA (2019). Effects of miniaturization in the anatomy of the minute springtail Mesaphorura sylvatica (Hexapoda: Collembola: Tullbergiidae). PeerJ..

[20] Büsse S, Gorb SN (2018). Material composition of the mouthpart cuticle in a damselfly larva (Insecta: Odonata) and its biomechanical significance. R Soc Open Sci.

[21] Favret C, Tzaud M, Erbe EF, Bauchan GR, Ochoa R (2016). An adhesive collophore may help direct the springtail jump. Ann Entomol Soc Am..

[22] Noble-Nesbitt J (1963). A site of water and ionic exchange with the medium in Podura aquatica L. (Collembola, Isotomidae). J. Exp. Biol..

[23] Schaller F (1970). Collembola (Springschwänze). Handbuch der Zoologie.

