# Supplementary figures and images for: On springtails (Hexapoda: Collembola): a morphofunctional study of the jumping apparatus
Source: Front Zool. 2022 Jul 29;19:21. doi: 10.1186/s12983-022-00463-y (PMC9336013; doi:10.1186/s12983-022-00463-y)

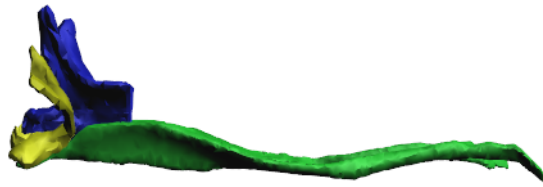

Supplement: Supplementary file 1 — Additional file 1. Fig 12. Interactive three-dimensional model of the movement of basal sclerites BR, BS2 and BS3. To activate the animation, click on the figure in Adobe Reader and rotate the object using the mouse. [file 12983_2022_463_MOESM1_ESM.pdf]
